# Drivers of firm-government engagement for technology ventures

**DOI:** 10.1371/journal.pone.0333710

**Published:** 2025-10-10

**Authors:** Lauren Lanahan, Iman Hemmatian, Amol M. Joshi, Evan E. Johnson

**Affiliations:** 1 Lundquist College of Business, University of Oregon, Eugene, Oregon, United States of America; 2 College of Business Administration, Cal Poly Pomona, Pomona, California, United States of America; 3 School of Business, Wake Forest University, Winston-Salem, North Carolina, United States of America; 4 Department of Public Policy, University of North Carolina at Chapel Hill, Chapel Hill, North Carolina, United States of America; UFSCar: Universidade Federal de Sao Carlos, BRAZIL

## Abstract

Prior scholarship generally examines the returns generated by firm-government engagement. These studies are based on an implicit and understudied assumption – the firm’s strategic choice of whether to engage with the government. Here, we unpack the drivers of this choice. To do so, we construct a population-level sample of U.S. high-tech ventures founded between 2015–2017; the full sample exceeds one million firms. We then utilize government records to identify *initial* firm-government engagement; approximately 24,000 high-tech ventures reveal this preference by firm age three. We examine a range of external and internal factors that may motivate such a choice. The results indicate that firm-government engagement most prominently coincides with firm resource constraints. Features driving such engagement include: (i) underrepresented minority-owned firms; (ii) small firms; (iii) firms with greater early-stage growth potential; and (iv) firms located in less intensive entrepreneurial settings. This study offers managerial, policy, and scholarly contributions by uncovering new insights around firm strategy and government opportunities for high-tech ventures.

*“We were a very young company. We had no clue on what to do or how to get started”,*

*The four partners spent the beginning years developing a plan for the company and contemplating if they would focus on the private or government sector. After seeking the help of the Florida Procurement Technical Assistance Center at the University of South Florida (USF), Matt Cetta, a founding partner of SBS (Strategic Business Systems), said…*

*“[We] chose government.”*

Small Business Development Centers (SBDC), Success Stories [1].

## Introduction

Technology ventures turn to the government to secure direct funding resources [2] and to access indirect benefits such as technical expertise [3,4], legitimacy [5–7], and market validation [8]. Uniquely, these firms tend to work on projects with outsized technical and time uncertainties [9], producing inherent high risk and increasing the likelihood of failure [10]. As a result, private financiers are more hesitant to provide the necessary financial resources [10–12].

To overcome these resource constraints and support the growth of young technology ventures, the U.S. federal government offers an array of public programs spanning local training and assistance, funding, and procurement contracting [13,14]. For example, in 2022, the U.S. federal government invested approximately $177 billion annually in R&D programs to support firms and market activity [4,15]. Ideally, public funding serves a distinct role because of the high-risk, uncertain nature of investments in basic scientific discovery [11,16,17]. In short, government investments persist given that the private sector lacks incentives to undertake these investments themselves due to the risk-return profile and time horizon [18,19].

Prior scholarship devotes considerable attention to examining the returns of these various government programs [14,20,21]. And depending on the outcome of interest – ranging from innovation to commercialization – the returns are mixed, putting to question whether government programs in fact serve as a complement or a substitute to market activity. On the one hand, numerous studies document favorable returns of firm-government engagement on innovation [22–24]. Yet, other studies find that these government programs can produce market distortions and decrease firm performance [25–28].

While the debate around the value of government returns persists, studies on both sides rely on an implicit and understudied assumption – the firm’s strategic choice of whether to engage with the government. In other words, despite the prevalence of government programs and corresponding scholarly attention examining firm output generated from public sector resources, such a context depends on the firm electing to seek these resources in the first place. However, somewhat surprisingly, no large-scale empirical studies directly examine the fundamental drivers of this strategic choice. Most often, scholars treat this firm-level decision as an underlying assumption and discuss the subsequent implications of this endogenous feature in the econometric model. This issue is most often addressed using matching techniques [29], regression discontinuity designs [30], selection models [27], or exploiting shifts in programmatic oversight [31]. The lack of prior research in understanding the underlying drivers of this relationship is problematic because the dearth of evidence can lead to missed opportunities for entrepreneurs and managers, ineffective policy design by policymakers, flawed theory development, underspecified research designs, and inconsistent recommendations by scholars.

To address these issues, we examine this core assumption by evaluating a range of antecedents that motivate young high-tech ventures to pursue *firm-government engagement*, which we formally define as *firm initiation and engagement with the government to seek resources.* These resources include funds available via contracts, grants, and loans, as well as training, mentoring, and other support. Specifically, our study investigates the following research question: what are the drivers of firm-government engagement for technology ventures?

To analyze this question, we construct a population-level sample of U.S. high-tech ventures founded between 2015–2017; the full sample exceeds one million firms. We then utilize government records to identify *initial* firm-government engagement. This comprises the sub-sample of approximately 24,000 high-tech ventures with a revealed preference in expressing any formal interest in establishing a relationship with the government over the firm’s first three years of operation.

To unpack the antecedents that plausibly drive this strategic firm choice, we examine a range of external and internal factors for the firm. The former includes three sets of ecosystem indicators: institutional intermediaries, capital infrastructure, and entrepreneurial intensity. Additionally, we include measures of political context and government opportunities. The latter set includes features of firm imprinting and early-stage growth.

The results are compelling and highlight salient trends that guide the strategic choice of firm-government engagement. First, the descriptive statistics reveal that approximately 2.3 percent of U.S. high-tech ventures establish such engagement. Second, the correlative regression results highlight that firm-government engagement appears to substitute for market opportunity. In other words, high-tech ventures with limited access to resources are more likely to seek such external engagement. Accordingly, we report that firms with underrepresented minority (URM) owners, small firms, and firms with greater early-stage growth potential (measured by credit and patent activity) each increase the likelihood to seek such engagement. Moreover, industries with heightened levels of federal contracting attract such engagement. Conversely, the substitutive effects most consistently include firms located in less intensive entrepreneurial settings, which lack adequate resources for ventures. This study offers managerial and scholarly contributions by uncovering insights around firm strategy and government opportunities for high-tech ventures seeking resources. The results provide empirical findings for guiding evidence-based policymaking by quantifying the extent to which government programs support ventures.

## Conceptual framing

Early-stage technology ventures engage with the federal government for various reasons. For example, it is well documented that ventures are resource-constrained and face inherent liabilities due to their “newness” and “smallness” [32–34]. Moreover, such uncertainty is amplified in high-tech sectors where the innovative frontier is constantly changing and accelerating [12]. Accordingly, innovation takes a long time to develop, and it has a high level of costs and risks coupled with persistent uncertainty [35]. Consequently, these challenges can hinder ventures’ survival and long-term growth [36,37].

Government programs offer one alternative for ventures at this juncture to traverse myriad early-stage challenges that include the infamous “valleys of death” [38]. Here, business operations are underway, and substantial costs are incurred but meaningful revenues have not yet materialized. Firms may seek government support to overcome these resource constraints as they pursue various growth opportunities in both private and public sector markets. Though the extensive research thus far on the relationship between government programs and market activity reveals mixed results. Some studies indicate complementarity of the programs, while others uncover substitution effects [39,40].

**Fig 1:**
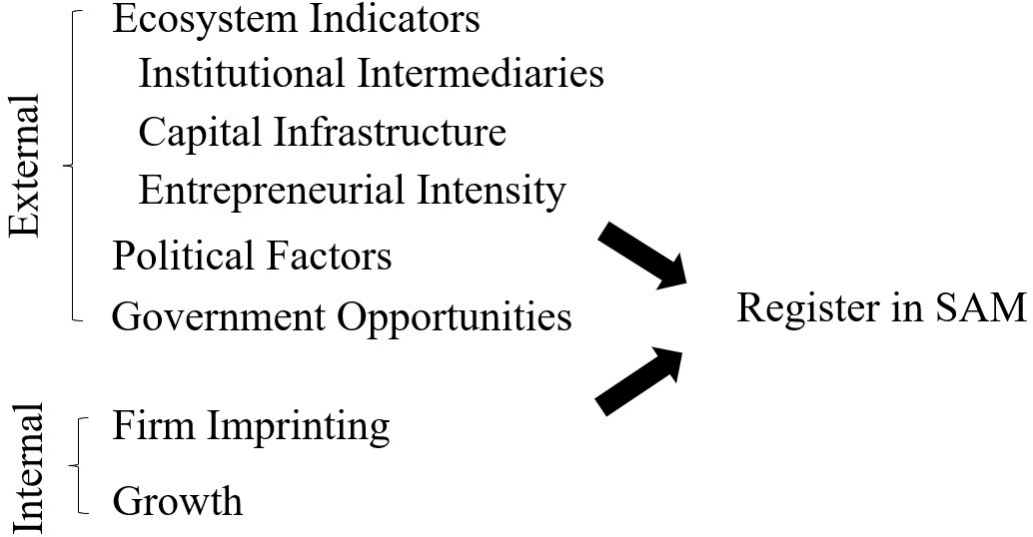
Conceptual framework. System for Award Management (SAM). By design, the administrative data repository of SAM is comprehensive and identifies the event when a firm elects to initiate engagement with the federal government as a potential grant recipient, supplier for procurement contracts, or borrower of loans.

To understand the antecedent factors that drive young high-tech ventures to establish an initial relationship with the government, we draw from literature streams on entrepreneurial ecosystems [41–43], resource dependency [29], entrepreneurship [44], and innovation [45]. Fig 1 illustrates our conceptual model, which in turn guides the empirical specification. In the context of young high-tech ventures, external and internal factors that influence firms to seek strategic engagement with the government remains an empirical question [46,47]. Ultimately, these features could be crucial predictors of long-term performance, growth, and survival of technology startups.

And to be clear, this study is exploratory. We do not present hypotheses or propositions articulating whether these various factors amplify or attenuate the strategic choice of the firm to engage with the government. Instead, we argue that internal and external factors place pressures (of varying degrees and directions) on firms that determine when and how young technology ventures strategically engage with federal government programs [48]. Moreover, this study is descriptive; we aim to understand whether and how various factors drive this choice. By design, we are unable to make causal claims as we are examining an endogenous process. In the following section, we define and review each feature under consideration.

### External factors

Entrepreneurial ecosystems consist of interconnected actors and elements that are organized to establish environments that support productive economic activity [49,50]. They play a central role in fostering technology-based ventures and defining the local landscape within which they operate [43,51,52]. These ecosystems involve complex interactions between young high-tech ventures, support organizations (e.g., universities, training institutes, and scientific parks), and other stakeholders (e.g., public sector and government entities) [53,54]. Drawing from this conceptual lens, we identify three ecosystem indicators that may motivate the venture’s choice to express formal interest in establishing a relationship with the government.

First, *institutional intermediaries* shape venture access to prominent organizations that serve as ecosystem-wide business leaders and advocates for the R&D enterprise [55–57]. For instance, when launching new high-tech ventures in a specific region, entrepreneurs establish mutually beneficial relationships with scientific communities to acquire knowledge and skilled human resources [58]. Research-active universities, incubators, and accelerators play a critical role in this regard not only by providing administrative and technical leadership, but also through extensive investments in human and physical capital [27,59,60]. Young high-tech ventures may use intermediaries, particularly research universities, to indirectly access government resources and knowledge networks [61,62]. By making these resources accessible, universities become vital hubs in supporting and fostering entrepreneurial ecosystems, especially in resource-constrained environments [63].

Second, *capital infrastructure* represents the breadth of financial capital that is accessible to ventures at their early stage [64]. Financial access is crucial for entrepreneurship and the development of entrepreneurial ecosystems [65]. This includes funding opportunities available via banks and local lending institutions [66] and the more competitive (and sizable) resources available via private financing [67]. Financial resources play a pivotal role in the entrepreneurial ecosystem and subsequent growth and innovation of technology ventures [50]. Availability of financial capital strengthens entrepreneurial ecosystems; in turn, strong ecosystems directly attract and efficiently allocate financial resources to new ventures [68,69]. Studies report that effective ecosystem development benefits from leveraging both government and private financial resources [70,71].

Third, *entrepreneurial intensity* accounts for the rates of startup activity and degree of market competition in an area. Heightened levels of such activity can bolster agglomeration benefits for ventures through common links of knowledge, capital, inputs, and demand [72,73]. High intensity enhances entrepreneurial ecosystems by attracting more talent and investment and encourages positive regional economic development and national policy reforms [74,75]. A high level of entrepreneurial activity is crucial for driving innovation, economic growth, and the overall health of entrepreneurial ecosystems [76,77]. In vibrant entrepreneurial ecosystems, entrepreneurial intensity signals market potential and attracts complementary resources and enhances knowledge sharing among ventures, especially in high-tech sectors where systemic innovation is prevalent [78,79].

In addition to the set of ecosystem indicators, political factors can play a significant role in shaping entrepreneurial ecosystem governance through policy formation and implementation [80]. This role is particularly important given that the resources are obtained through firm-government interactions. Research reports that firms maintain alignment with changing government policies and ecosystem dynamics through adaptive approaches [81]. *Political context* comprises both the political ideology in the local region and alignment between local and national politics; both may influence the extent to which ventures consider engagement with the U.S. federal government [52]. Moreover, government support for ventures and technology is not only guided by national efforts, but also public support is locally tailored via state government policies and regional initiatives [53,82]. Overall, politics plays a significant role in shaping entrepreneurial ecosystems, where political dynamics and government policies can foster or hinder entrepreneurial activity [83,84].

Lastly, we consider *government opportunities*. Government spending programs help support entrepreneurial ecosystems by offering funding, infrastructure, education, and policy measures [51]. This includes regulated programs designed to bolster resource access to new ventures [51]. Young high-tech ventures may strategically utilize specific government programs to support their innovation, growth, and survival [2]. Additionally, certain industries are of greater interest to government projects and initiatives [22]. U.S. government contracting is an immense enterprise. For example, in 2019, the U.S. Department of Defense spent 403.9 billion USD on procurement contracts with firms. This level of activity represents approximately 73 percent of the agency’s total annual spending, or nearly 2 percent of U.S. GDP [85]. Trends in government contracting likely shape opportunities and may motivate ventures to develop a relationship with the government.

### Internal factors

We draw from the entrepreneurship, resource dependency, and innovation literature streams to identify internal factors of firm-level imprinting [29,86,87] and early-stage growth potential [88]. We argue such features are likely to motivate a venture’s choice to express formal interest in establishing a relationship with the government. *Firm imprinting* includes founder demographics [20,44], whereas *growth* captures the early potential for a venture [89].

To elaborate on the former, studies report that firm founders’ demographic backgrounds and prior experiences strongly influence a technology venture’s propensity to pursue federal government opportunities [90]. These characteristics and past professional experiences impact whether entrepreneurs perceive interaction with the government as beneficial, accessible, or aligned with their venture goals [91,92]. As a salient example, the Small Business Administration (SBA) procurement scorecard is an annual assessment that measures how well federal agencies achieve small business contracting goals set forth by government statute [13]. Each year, a portion of both the prime and subcontracting goals are set for minority-owned, women-owned, or veteran-owned businesses. Minority founders or those who have previously served in the military often view government engagement as a familiar process and are more likely to establish the relationship from the start.

As for the latter, early-state growth orientation likely affects whether firms elect to engage in government opportunities as well. New ventures often face resource constraints that make them dependent on external support, such as government programs [93,94]. Of note, the size (and age) of the firm is correlated with resource constraints [89,95]. From the resource dependency perspective, this relationship shapes how new ventures operate, survive, grow or strategize [96–98]. The venture’s initial resource endowment likely impacts their decision to solicit external resources. When a firm heavily depends on specialized funding sources or third-party validation that private investors hesitate to offer, government programs like the Small Business Innovation Research program become attractive partners because they provide non-dilutive R&D funding, legitimacy, and/or technical expertise that can potentially unlock subsequent private financing [6,51]. Additionally, patenting defines a strategic role among high-tech endeavors as firms seek to offset the high risk and uncertainty tied to innovative pursuits with formalized contracting [45]. Patenting signals the novelty, usefulness, and commercial potential of a venture’s intellectual property and may demonstrate the venture’s ability to mitigate and overcome technical risks [99]. Recent scholarship is beginning to uncover differential positioning for young and small firms engaged in patenting endeavors, reporting a unique role of government in supporting such activity [100]. The extent to which a young high-tech venture is resource-constrained – based on internal strengths and capabilities – plausibly influences whether it seeks external government engagement [29].

## Empirical context

### Sample and key dependent variable

Directly answering our research question requires that we begin with a population-level sample of U.S. young high-tech ventures. Thus, we draw from the full repository of establishments listed in the National Establishment Times Series (NETS) database. NETS is collected by Dun & Bradstreet, which traces credit activity in the U.S. This proprietary source includes over 82.4 million U.S. establishments from 1990–2020 and reports annual detail on a range of characteristics that include location, industry, and performance measures. This database has been used by scholars [101,102] and has been found to provide reasonable estimates compared to restricted administrative government records of U.S. firm activity [103].

The sample of targeted firms share the following features: (i) U.S. owned; (ii) operate in high-tech industry sectors as defined by the Bureau of Labor Statistics [104]; (iii) are single-establishment organizations; and (iv) are founded between 2015–2017. Regarding the last specification, we set this timeframe due to the following. First, 2015 defines the initial year that the administrative source to discern whether firms express interest with the federal government (i.e., as reported in the U.S. General Services Administration’s System for Award Management (SAM)) is publicly available. Second, 2017 defines the last year to trace firms over a standardized three-year window that precedes the outbreak of COVID-19. This global event introduced unique confounding factors driving interest in government engagement that lie outside the scope of this analysis [105]. Altogether, this defines a total population of 1,014,868 unique firms nationwide.

To validate the completeness of NETS, we compare the level of single-establishment ventures reported in NETS to new registrations reported in every Secretary of State (SoS) repository over a comparable timeframe (2015–2017). This includes ventures in all industries (i.e., beyond high-tech industry sectors). Overall, NETS reports detail for 88 percent of U.S. startups reported in the SoS repository. This result attests to the completeness of the NETS data source and its suitability for our research design. And for further insight into the sample, approximately 12 percent operate in high-tech industry sectors. To illustrate regional variation, we report statistics by state in Table 1 (Columns 1 and 2).

**Table 1:** Sample verification.

	Column 1			Column 2		Column 3	
	U.S. Ventures by various source			U.S. High-tech Ventures in sample		U.S. High-tech Ventures in SAM	
	NETS	SoS	Ratio (NETS/SoS)	NETS	Ratio (HT/Total)	SAM	Ratio (SAM/NETS)
AK	13,700	20,808	(0.66)	1,768	(0.13)	106	(0.06)
AL	96,349	83,968	(1.15)	8,232	(0.09)	322	(0.04)
AR	41,631	59,655	(0.70)	3,983	(0.10)	96	(0.02)
AZ	309,360	211,174	(1.46)	45,028	(0.15)	478	(0.01)
CA	965,501	755,826	(1.28)	132,795	(0.14)	2462	(0.02)
CO	248,654	324,007	(0.77)	35,144	(0.14)	713	(0.02)
CT	102,988	91,858	(1.12)	13,365	(0.13)	138	(0.01)
DC	19,532	37,584	(0.52)	3,987	(0.20)	551	(0.14)
DE	20,566	546,555	(0.04)	3,599	(0.17)	135	(0.04)
FL	937,173	1,107,287	(0.85)	100,603	(0.11)	1658	(0.02)
GA	317,475	362,603	(0.88)	36,413	(0.11)	957	(0.03)
HI	34,127	48,461	(0.70)	4,238	(0.12)	124	(0.03)
IA	72,903	65,665	(1.11)	7,225	(0.10)	92	(0.01)
ID	56,534	60,661	(0.93)	6,598	(0.12)	141	(0.02)
IL	234,723	256,267	(0.92)	29,253	(0.12)	534	(0.02)
IN	142,275	164,693	(0.86)	15,267	(0.11)	233	(0.02)
KS	43,256	62,457	(0.69)	5,048	(0.12)	139	(0.03)
KY	88,696	80,684	(1.10)	8,358	(0.09)	123	(0.01)
LA	145,736	135,156	(1.08)	10,771	(0.07)	270	(0.03)
MA	160,598	131,959	(1.22)	21,948	(0.14)	533	(0.02)
MD	178,207	186,367	(0.96)	24,843	(0.14)	1926	(0.08)
ME	22,731	28,458	(0.80)	2,595	(0.11)	59	(0.02)
MI	175,946	256,142	(0.69)	22,691	(0.13)	412	(0.02)
MN	139,425	141,617	(0.98)	17,435	(0.13)	204	(0.01)
MO	154,338	179,809	(0.86)	14,886	(0.10)	271	(0.02)
MS	83,103	74,891	(1.11)	5,321	(0.06)	113	(0.02)
MT	34,306	55,118	(0.62)	3,947	(0.12)	104	(0.03)
NC	228,953	226,534	(1.01)	26,303	(0.11)	654	(0.02)
ND	19,562	25,444	(0.77)	1,931	(0.10)	43	(0.02)
NE	40,486	41,363	(0.98)	4,073	(0.10)	90	(0.02)
NH	39,723	37,754	(1.05)	5,505	(0.14)	89	(0.02)
NJ	249,205	312,004	(0.80)	32,865	(0.13)	416	(0.01)
NM	39,955	52,535	(0.76)	5,647	(0.14)	241	(0.04)
NV	61,982	161,336	(0.38)	8,355	(0.13)	229	(0.03)
NY	445,748	555,705	(0.80)	58,302	(0.13)	863	(0.01)
OH	209,763	257,635	(0.81)	23,575	(0.11)	467	(0.02)
OK	90,695	111,030	(0.82)	10,250	(0.11)	197	(0.02)
OR	123,807	138,441	(0.89)	14,907	(0.12)	277	(0.02)
PA	210,408	237,886	(0.88)	25,779	(0.12)	604	(0.02)
PR	6,062	42,710	(0.14)	918	(0.15)	123	(0.13)
RI	26,142	27,689	(0.94)	3,157	(0.12)	56	(0.02)
SC	117,292	137,985	(0.85)	11,475	(0.10)	259	(0.02)
SD	22,104	23,498	(0.94)	2,295	(0.10)	49	(0.02)
TN	151,873	105,620	(1.44)	14,343	(0.09)	305	(0.02)
TX	754,656	625,545	(1.21)	83,758	(0.11)	1631	(0.02)
UT	120,561	153,087	(0.79)	15,329	(0.13)	203	(0.01)
VA	214,491	245,362	(0.87)	32,426	(0.15)	2928	(0.09)
VI	580	5,633	(0.10)	75	(0.13)	2	(0.03)
VT	16,067	22,228	(0.72)	2,012	(0.13)	48	(0.02)
WA	233,249	202,134	(1.15)	31,311	(0.13)	560	(0.02)
WI	88,941	129,703	(0.69)	10,221	(0.11)	210	(0.02)
WV	26,624	47,384	(0.56)	2,854	(0.11)	81	(0.03)
WY	11,153	92,923	(0.12)	1,861	(0.17)	84	(0.05)
**Total**	**8,389,915**	**9,548,898**	**(0.88)**	**1,014,868**	**(0.12)**	**23,603**	**(0.02)**

Table reports aggregate count of venture activity based on firm founding from 2015–2017 for 50 U.S. states, Washington DC, Puerto Rico, and the U.S. Virgin Islands. For SoS, we collect business registration records from the SoS website of each state or territory. For NETS, we derive the sample of U.S. ventures based on ownership (i.e., U.S. owned), age (founded 2015–2017), and single-establishment organization. Column 1 reports venture ratio between the two sources: NETS/SoS; Column 2 reports high-tech venture ratio based on NETS detail (U.S. high-tech ventures/total ventures); Column 3 reports high-tech venture ratio with firm-government engagement based on SAM detail (U.S. high-tech ventures listed in SAM/total U.S. high-tech ventures listed in NETS).

As previously mentioned, we draw upon the U.S. General Services Administration’s System for Award Management (SAM) database to identify the population of firms with an *expressed interest* in transacting with the U.S. federal government [106]. By design, this administrative data repository is comprehensive and identifies the event when a firm elects to initiate engagement with the federal government as a potential grant recipient, supplier, or borrower. Effectively, registration in SAM is a *revealed preference*, which is the initial strategic “stepping stone” for firms to engage in federal programs by either participating in training or assistance, applying for R&D subsidies, or seeking contracting opportunities. (In other words, this includes all firm applicants seeking such government services and those that ultimately secure the resources.)

Table 2 details a range of government programs firms can access after registering in SAM. The Small Business Administration (SBA) leads these efforts, serving as a federal executive branch agency to support entrepreneurs and small businesses and help them start, build, and grow businesses. The SBA offers a variety of services, including financial assistance, counseling, and contracting opportunities. The programs are designed to provide comprehensive support to small businesses at various stages of their growth and development.

**Table 2:** U.S. SBA programs and type(s).

	Program	Description
**Local Training and Assistance**	Federal Contracting Assistance	Procurement Technical Assistance Centers (PTAC) provides technical assistance to businesses interested in selling products or services to federal, state, and local governments.
	Export Finance Managers	Small Business Administration (SBA) works directly with small business exporters through U.S. Export Assistance Centers (USEACs), which are co-located with the Department of Commerce and the Export-Import Bank of the United States.
	U.S. Export Assistance Centers	U.S. Export Assistance Centers (USEACs) support American small businesses who want to compete globally by exporting.
	Washington Export Outreach Team	Established in 2013 following a national mandate to do more to support international trade, the Washington Export Outreach Team (WEOT) is a collaborative team of local, state, regional, and federal export assistance agencies who provide you the tools and resources you need to sell internationally.
	Utah International Outreach Team	Established in 2020, the Utah International Outreach Team (UIOT) is a collaborative team of local, state, regional, and federal export assistance agencies providing exporters with the resources they need to sell internationally.
	Small Business Development Centers (SBDC)	Small Business Development Centers (SBDCs) provide counseling and training to small businesses including working with the SBA to develop and provide informational tools to support business start-ups and existing business expansion.
	SCORE Business Mentoring	SCORE, the nation’s largest network of volunteer, expert business mentors, is dedicated to helping small businesses plan, launch, manage and grow.
	Veterans Business Outreach Center (VBOC) program	The Veterans Business Outreach Center (VBOC) program is designed to provide entrepreneurial development services such as business training, counseling, and resource partner referrals to transitioning service members, veterans, National Guard & Reserve members, and military spouses interested in starting or growing a small business. SBA has 22 organizations participating in this cooperative agreement and serving as VBOCs.
	Women’s Business Centers	Women’s Business Centers (WBCs) are a part a national network of entrepreneurship centers throughout the United States and its territories, which are designed to assist women in starting and growing small businesses. WBCs seek to “level the playing field” for women entrepreneurs, who still face unique obstacles in the business world.
	Community Navigators	The Community Navigator Pilot Program is an American Rescue Plan initiative designed to reduce barriers that underrepresented and underserved entrepreneurs often face in accessing the programs they need to recover, grow, or start their businesses. The program will provide a total of $100 million in funding to 51 organizations that will work with hundreds of local community groups to improve access to SBA and government resources for America’s entrepreneurs.
	Regional Innovation Clusters	Regional Innovation Clusters are geographically concentrated networks of small businesses, suppliers, service providers, and related institutions that work together to maximize their strengths and resources, allowing them to compete on a larger scale, and drive innovation and job creation.
	Veterans Business Development Officers	Veterans Business Development Officers work in each of SBA’s 68 District Offices, and are familiar with federal, state, and local programs that assist veteran entrepreneurs.
**Funding**	Small Business Innovation Research (SBIR)	Small Business Innovation Research (SBIR) program encourages small businesses to engage in Federal Research/Research and Development (R/R&D) with the potential for commercialization. SBA serves as the coordinating agency for the SBIR program. The SBIR program is structured in three phases. Phase I awards are generally $50,000 - $250,000 for 6 months. Phase II awards are generally $750,000 for 2 years. The SBIR program does not fund Phase III.
	Small Business Technology Transfer (STTR)	Small Business Technology Transfer (STTR) program encourages small businesses to engage in Federal Research/Research and Development (R/R&D) with the potential for commercialization. SBA serves as the coordinating agency for the STTR program. STTR project requires the small business to be teamed with a non-profit research institution. Also, the STTR program is focused on transferring technology from the research institution to the small business and ultimately to the market. The STTR program is structured in three phases. Phase I awards are generally $50,000 - $250,000 for 1 year. Phase II awards are generally $750,000 for 2 years. The STTR program does not fund Phase III.
	7(a) loans	The 7(a) Loan Program, SBA’s most common loan program, includes financial help for small businesses with special requirements. This is the best option when real estate is part of a business purchase, but it can also be used for: (1) Short- and long-term working capital; (2) Refinance current business debt; (3) Purchase furniture, fixtures, and supplies. The maximum loan amount is $5 million.
	504 loans	The CDC/504 Loan Program provides long-term, fixed rate financing for major fixed assets that promote business growth and job creation. The maximum loan amount is $5 million.
	Microloans	The microloan program provides loans up to $50,000 to help small businesses and certain not-for-profit childcare centers start up and expand.
	Investment capital	Small businesses find an investor for their business through a Small Business Investment Company (SBIC) licensed by SBA.
	Physical damage loans	Homeowners, renters, nonprofit organizations, and businesses of all sizes are eligible to apply for physical disaster assistance. The maximum loan amount is $200,000.
	Mitigation assistance	SBA offers low-interest disaster loans to homeowners and small businesses impacted by declared natural and other disasters. Eligible SBA disaster loan borrowers may choose to receive expanded funding to help mitigate their home or business against future disasters. SBA disaster loans can be increased up to 20% to make building upgrades.
	Economic injury disaster loans	SBA offers Economic Injury Disaster Loan (EIDL) to small businesses, small agricultural cooperatives, and most private nonprofit organizations located in a declared disaster area and which have suffered substantial economic injury. The maximum loan amount is $2 million.
	Military reservist loan	SBA provides loans to help eligible small businesses with operating expenses if they have an essential employee who is a military reservist called to active duty. The maximum loan amount is $2 million.
	Surety bonds	Surety bonds help small businesses win contracts by providing customer with a guarantee that the work will be completed. Many public and private contracts require surety bonds, these are offered by surety companies.
	State Trade Expansion Program	STEP provides financial awards to state and territory governments to assist small businesses with export development.
	Grants for community organizations	SBA offers grants for community organizations and cooperative agreements that support small business growth and development.
**Federal Contracting**	Small Disadvantaged Business	Each year, the Federal Government awards about 10% of all federal contract dollars, or roughly $50 billion in contracts, to Small Disadvantaged Businesses.
	Women-Owned Small Business Federal Contracting Program	To help provide a level playing field for women business owners, the government limits competition for certain contracts to businesses that participate in the Women-Owned Small Business (WOSB) Federal Contracting program.
	Veteran assistance programs	Every year, the federal government awards a portion of contracting dollars specifically to businesses owned by veterans. Also, small businesses owned by veterans may be eligible to purchase surplus property from the federal government.
	8(a) Business Development program	The 8(a) program is a robust nine-year program created to help firms owned and controlled by socially and economically disadvantaged individuals. The federal government’s goal is to award at least 5% of all federal contracting dollars to small, disadvantaged businesses each year.
	SBA Mentor-Protégé program	SBA helps eligible small businesses (protégés) gain capacity and win government contracts through partnerships with more experienced companies (mentors).
	Joint ventures	Joint ventures allow certain businesses to compete together for government contracts reserved for small businesses.
	7(j) Management and Technical Assistance program	SBA provides high-quality assistance to SBA-approved small businesses to help them successfully compete for federal, state, and local contracting opportunities as a prime or subcontractor.
	HUBZone program	SBA helps small businesses in urban and rural communities gain preferential access to federal procurement opportunities. The government limits competition for certain contracts to businesses in historically underutilized business zones. The goal is awarding at least 3% of federal contract dollars to HUBZone-certified companies each year.
	Natural Resource Sales Assistance program	SBA uses small business set-asides to help them get a fair share of government property sales and leases.

Source is SBA website: https://www.sba.gov (Accessed September 25, 2022).

All entities registered in NETS and SAM are required to obtain a unique DUNS Number (Data Universal Numbering System) from Dun & Bradstreet. We use this 9-digit identifier to match firms precisely across each source. Based on founding dates, 92.9 percent of firms listed in SAM matched the NETS sample. Moreover, to examine strategic behavior at an early stage, we identify registration in SAM by firm age three for the entire population of U.S. high-tech ventures. The three-year timing specification both defines a standardized window to avoid right-censoring and precedes the global outbreak of COVID-19. Nationally, 2.3 percent of U.S. high-tech ventures (23,603 firms) register in SAM in this timeframe; we report trends by state (Table 1 Column 3).

**Fig 2:**
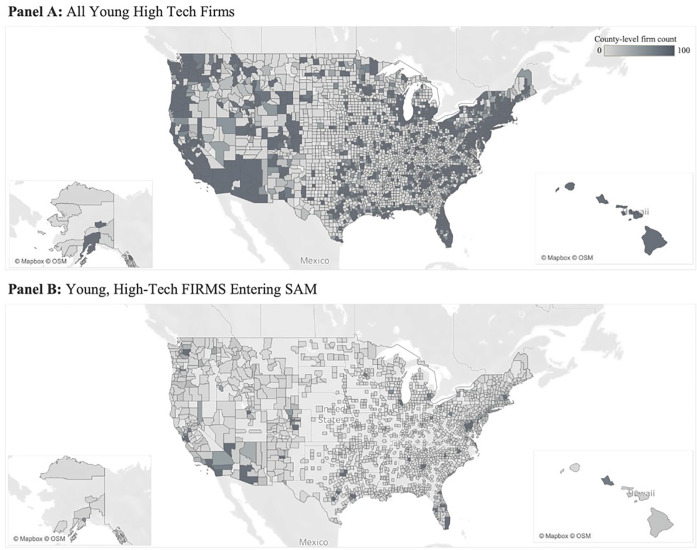
Geographic distribution of U.S. high-tech ventures and firm-government engagement. Maps illustrate aggregate count of U.S. high-tech ventures by FIPS (Federal Information Processing Standards county code) founded from 2015–2017. Panel A reports the population of high-tech ventures (1,014,868 firms); Panel B reports high-tech ventures entering SAM by firm age 3 (23,603 firms). This map is based on Mapbox tools and data, with OpenStreetMap. It was made using Tableau Desktop Software v. 2024.1.

Fig 2 illustrates the geographic distribution of this sample by county. Panel A depicts the distribution for the population of U.S. high-tech ventures (1,014,868 firms); Panel B depicts the sub-sample that register in SAM by firm age three (23,603 firms). These images illustrate the geographic reach across the U.S. (Panel A correlates highly with population density); however, Panel B depicts the concentration (or lack thereof) of such firm-government engagement in certain areas. The metropolitan area around the national capital accounts for a disproportionate amount of firm-government engagement; we confirm this with the leading trends for DC, VA, and MD reported in Table 1 (column 3).

### Regressors

Beginning with external factors, we construct the set of ecosystem indicators by leveraging features of geography. Importantly, high-tech activity is especially reliant on spillovers [24,107] and agglomeration economies [108–110]. These valuable externalities have essential locational components. For example, it is well documented that knowledge spillovers are most pronounced within short geographic spans (i.e., within a given county) and that local industry structures impact access to common resources such as labor pools and supply chains [111,112]. We leverage geographic proximity to various institutions as single-establishment ventures are likely to be especially reliant on their local surroundings. Contrastingly, older firms (which we exclude from this study) are less constrained by their proximate geography given their greater access to resources and multi-establishment positioning (e.g., setting up branch offices to serve new customers).

For *institutional intermediaries*, we identify ventures located within the median sample distance to their proximate university and accelerator. To derive this indicator, we rely on the locations of two comprehensive lists of U.S. organizations – universities designated as research-active by the Carnegie Classification of Institutions of Higher Education and accelerators [113]. Based on the physical address of the firm and organization set, we compute the nearest distance (in kilometers) between each firm in the sample and each organization type (i.e., nearest distance between venture and university and nearest distance between venture and accelerator). We assign a value of one to ventures with closer proximity to both universities and accelerators as we assume they have greater access to these institutions.

We apply a similar methodology for *capital infrastructure*. Here, we identify ventures located within the median sample distance to their proximate bank or local lending institution, angel investor, and venture capital organization. To derive this indicator, we rely on the locations of entities in three comprehensive lists of U.S. organizations – Federal Deposit Insurance Corporations (FDIC), angel investors, and venture capital (VC) organizations.

For *entrepreneurial intensity*, we identify ventures located in counties with startup ratios exceeding the sample mean and in counties with unconcentrated markets. We derive the latter from the Herfindahl-Hirschman index, which is based on country trends of firm sales by 3-digit North American Industry Classification System (NAICS) (i.e., HHI < 0.15). We assign a value of one to ventures located in areas with higher rates of startup activity and greater market competition (i.e., less market concentration and consolidation).

For *political context,* we construct two measures based on local and national voting patterns using the Dave Leip Atlas [114]. We identify Democratic counties based on the majority vote from recent national elections in alignment with the panel years at firm founding (Presidential and U.S. Senate). We also construct a binary indicator to identify alignment (or not) between local county voting patterns and national outcomes. Moreover, we include an indicator tracing state government technology-based economic development (TBED) policy activity to account for more localized government effects [53].

For *government opportunity* we construct two indicators based on venture proximity to U.S federal Procurement Technical Assistance Centers (PTAC) and Community Development Financial Institutions (CDFI), respectively. These organizations serve as government regulated training programs and account for access to publicly funded training opportunities [51,115]. Lastly, we identify the set of industries with disproportionate government contracting activity. Using comprehensive administrative records reported in the Federal Procurement Data System (FPDS), we identify the top decile of investments made to high-technology industries and the top decile of investments made to small businesses in 2015. For the latter, we leverage size rather than age as FPDS does not report details on establishment age; nevertheless, both age and size are correlated [116,117]. Together, this set defines the selection of industries most salient to the core research question around firm-government engagement for young high-tech ventures.

For internal factors of *firm imprinting*, we account for demographic features of the founder by identifying URM-owned – and, separately, woman- or minority-owned – firms. Additionally, we include several measures of growth. Using firm-level data on employment, we construct a binary indicator to distinguish larger from smaller ventures. Moreover, using firm-level records from Dun & Bradstreet (and subsequently reported in NETS), we include an indicator to identify whether the venture established any credit with a lender or supplier. We define both measures based on activity by firm age two. Separately, we identify the sample of ventures with any patenting activity by firm age three. For this measure, we match the population of ventures in the full sample to the corpus of assignees in USPTO (PatentsView) over the timeframe of interest (2015–2019). We use common text processing approaches in cleaning, standardizing, and matching firm name to define matches as those with greater than 0.95 similarity. Table 3 reports detail for the full set of variables, timing specification, sources, and functional forms.

**Table 3:** Detail and data source for DV and regressors.

Variables	Timing	Detail; Source
Firm Register in SAM	By Age 3	Government Accounting Office System for Awards Management (SAM, binary)
Internal Regressors		
URM Owned	Founding	NETS (under-represented woman and/or minority owned, binary)
Woman Owned	Founding	NETS (binary)
Minority Owned	Founding	NETS (binary)
Size	By Age 2	NETS (binary, FTE > 1)
Any Credit	By Age 2	NETS (binary)
Any Patent	By Age 3	USPTO (Homepage 2025 | PatentsView); fuzzy string match algorithm based on founding year and firm name (exclude matches < 0.95 similarity)
External Regressors		
Ecosystem Indicators		
Institutional Intermediaries	Founding	Venture geographically located within the *median* sample distance to the proximate university and accelerator (binary); University organization list from NCSES (Carnegie Classification of research active higher education institutions); accelerator organization list from Pitchbook
Capital Infrastructure	Founding	Venture geographically located within the *median* sample distance to the proximate FDIC, Angel, and VC (binary); FDIC organization list from (https://banks.data.fdic.gov/docs/); angel and VC organization list from Pitchbook
Entrepreneurial Intensity	Founding	Venture located in county with startup ratio exceeding the sample mean and HHI < 0.15 (i.e., unconcentrated market) (binary); HHI derived from firm sales by county and NAICS 3-digit
Political		
Alignment	Founding	Dave Leip Atlas (DLA); county & national political alignment (binary: align = 1; not align = 0). Based on 2014 senate or 2012 presidential race for firms founded in 2015; based on 2016 presidential race for ventures founded 2016 and 2017
Democratic County	Founding	DLA; democratic votes exceed republican votes (binary). Based on 2014 senate or 2012 presidential race for firms founded in 2015; based on 2016 presidential race for ventures founded 2016 and 2017
Government Training		
PTAC	Founding	Venture geographically located within the *median* sample distance to the proximate PTAC (binary); PTAC organization list from Association of Procurement Technical Centers (https://www.aptac-us.org/find-a-ptac/)
CDFI	Founding	Venture geographically located within the *median* sample distance to the proximate CDFI (binary); CDFI organization list from U.S. Department of Treasury Community Development Financial Institutions Fund (https://tinyurl.com/259pa3ea)
Additional Moderators		
TBED	Founding	Dummy indicator of presence of US state technology-based economic development (TBED) policy based on year of venture founding
FPDS	Founding	Dummy indicator based on government contracting investment in areas of high-tech and small organizations. We identify the set of 6-digit NAICS codes with Federal Procurement Data System (FPDS) in leading decile in 2015 for high-tech industries and small organizations. (Federal Procurement Data System – Next Generation)

Median sample distances based on U.S. high-tech venture sample reported in NETS. Table 5 reports median distances (km).

### Descriptive statistics

Table 4 reports descriptive statistics along with comparison of means and t-tests for the primary set of variables. We report descriptive statistics and comparison of means for raw measures in Table 5. In both tables, the comparison of means reports differences between the full sample of U.S. high-tech ventures to the subset that registered in SAM.

**Table 4:** Descriptive statistics of DV and regressors.

	Mean	S.D.	U.S. high-tech ventures	Sub-set reg. in SAM	t-stat	
DV: Register in SAM by Age 3	0.02	0.15	0.00	1.00	.	
Internal Regressors						
Firm Imprinting						
URM	0.05	0.23	0.04	0.54	−152.93	***
Woman	0.04	0.20	0.04	0.36	−102.66	***
Minority	0.02	0.14	0.01	0.35	−108.39	***
Growth						
Size (FTE > 1)	0.84	0.37	0.84	0.61	72.46	***
Any Credit	0.02	0.13	0.02	0.02	−7.97	***
Any Patent	0.01	0.08	0.01	0.02	−11.46	***
External Regressors						
Ecosystem (& interactions)						
Institutional	0.30	0.46	0.30	0.36	−19.17	***
Capital	0.29	0.45	0.29	0.30	−3.48	***
Entrepreneurial (Ent.)	0.46	0.50	0.46	0.42	13.31	***
Institutional * Capital	0.17	0.37	0.17	0.20	−11.37	***
Capital * Entrepreneurial	0.16	0.37	0.16	0.14	7.95	***
Institutional * Entrepreneurial	0.16	0.37	0.16	0.18	−7.14	***
Institutional * Capital * Ent.	0.09	0.29	0.09	0.10	−2.21	**
Political Factors						
Political Alignment	0.43	0.50	0.43	0.40	10.65	***
Democratic County	0.63	0.48	0.63	0.66	−11.82	***
Government Training						
PTAC	0.50	0.50	0.50	0.54	−12.39	***
CDFI	0.50	0.50	0.50	0.37	40.24	***
Additional Moderators						
TBED	0.95	0.22	0.95	0.93	10.28	***
FPDS	0.53	0.50	0.53	0.77	−85.44	***
Unique Firm Observations	1,014,868		991,265	23,603		

Comparison of means reports between full sample and sub-sample that register in SAM by age 3. Entrepreneurial access measures reported for 1,014,115 observations. Political factors reported for 1,012,110 observations.

**Table 5:** Additional descriptive statistics, including raw measures.

	Mean	S.D.	Median	25th	75th	Min.	Max.	Control	Treat	t-stat	
Founding Year 2015	0.27	0.44	0.00	0.00	1.00	0.00	1.00	0.26	0.33	−22.32	***
Founding Year 2016	0.37	0.48	0.00	0.00	1.00	0.00	1.00	0.37	0.36	4.22	***
Founding Year 2017	0.36	0.48	0.00	0.00	1.00	0.00	1.00	0.36	0.31	18.37	***
Employment (FTE)	2.56	1.56	2.00	2.00	3.00	1.00	10.00	2.56	2.59	−1.56	
Sales ($100k, adjusted)	2.34	2.35	1.65	1.13	2.64	0.00	16.95	2.33	2.68	−15.57	***
Distance (km) from venture to nearest…											
University	81.47	84.76	47.42	15.68	129.02	0.02	383.00	81.67	73.13	15.14	***
Accelerator	30.58	51.15	12.46	4.89	29.87	0.00	312.67	30.59	29.87	2.03	**
FDIC	1.61	2.02	0.96	0.41	1.89	0.00	11.67	1.60	1.68	−5.93	***
Angel	19.97	37.16	5.79	2.02	17.37	0.00	207.68	20.00	18.55	6.09	***
VC	18.16	32.12	5.86	2.04	16.72	0.00	183.03	18.19	16.96	5.90	***
PTAC	56.03	53.75	35.44	15.12	84.22	0.00	235.71	56.08	53.81	6.34	***
CDFI	283.83	149.90	266.29	165.55	392.41	0.80	660.18	282.92	321.95	−39.19	***
Herfindahl-Hirschman Index											
Unconcentrated (< 0.15)	0.91	0.28	1.00	1.00	1.00	0.00	1.00	0.91	0.90	5.43	***
Moderate (0.15–0.25)	0.04	0.20	0.00	0.00	0.00	0.00	1.00	0.04	0.04	−1.21	
Concentrated (> 0.25)	0.05	0.22	0.00	0.00	0.00	0.00	1.00	0.05	0.06	−5.91	***
Startup ratio (Ventures_ct_/Total Firm_ct_)	0.12	0.03	0.11	0.10	0.13	0.01	0.25	0.12	0.11	27.30	***
URM ratio (URM Ventures_zt_/Ventures_zt_)	0.04	0.03	0.03	0.02	0.04	0.00	1.00	0.03	0.05	−48.74	***
SAM ratio (SAM Venture_zt_/Ventures_zt_; lagged 1 yr)	0.01	0.02	0.01	0.00	0.02	0.00	0.10	0.01	0.02	−63.30	***
California	0.13	0.34	0.00	0.00	0.00	0.00	1.00	0.13	0.10	13.46	***
Florida	0.10	0.30	0.00	0.00	0.00	0.00	1.00	0.10	0.07	17.49	***
5416- (Mgmt, Sci, Tech. Consulting)	0.29	0.46	0.00	0.00	1.00	0.00	1.00	0.29	0.38	−27.42	***
5414- (Specialized Design Services)	0.12	0.33	0.00	0.00	0.00	0.00	1.00	0.12	0.03	89.65	***
Unique Firm Observations	1,014,868							991,265	23,603		

Comparison of means reports between full sample and sub-sample that register in SAM by age 3. All continuous measures are winsorized (1^st^ and 99^th^). Entrepreneurial access and Herfindahl-Hirschman Index reported for 1,014,115 observations. *c* denotes county designation; *z* denotes zip code designation.

The most substantial observation is the consistent trend of differences in means. Comparing high-tech ventures that register in SAM to the full sample of U.S. high-tech ventures, the former set is substantially more likely to be URM-owned, smaller in size, and more likely to patent. In terms of external factors, ventures that register in SAM have greater access to institutional intermediaries and capital infrastructure, yet they are in areas with less entrepreneurial intensity. They are more likely to be in Democratic-leaning counties; however, local voting is less likely to be aligned with national political control. They are more proximate to PTACs and further from CDFIs. Lastly, while prevalent overall, they are slightly less likely to be in states with TBED policies, and they operate in industries that receive disproportionate support via government contracting.

S1 Table reports the results from the correlation matrix. Panel A reports the set of correlations for the primary variables (reported in Table 4), and Panel B reports the correlations for the measures with the raw functional form (reported in Table 5). Across all, correlations are limited, suggesting that multi-collinearity is not a concern [118].

## Research design

Fundamentally, we aim to understand factors driving the choice made by firms to initiate and engage with the U.S. federal government. We set up a maximum likelihood logistic regression to assess the relationship of a series of external and internal antecedent factors on the dependent variable, *Register in SAM*. We express this relationship in Equation 1:


Pr(Y=1)= f(Internal, External, Dummies, ε) (1)

The data are structured in cross-sectional format, yet they account for dynamic activity over the initial three years of the firm’s operation. We do not execute a firm fixed effect model given that most regressors of interest are time-invariant. Specifically, the external and internal regressors are based on static measures (most at founding, though growth indicators are based on activity at firm age two or three), while the dependent variable captures activity by firm age three. In addition to the set of ecosystem indicators, we also include their interactions. Moreover, we include an exhaustive set of year, state, and industry dummies (i.e., 4-digit NAICS, representing industry group) based on firm founding details. Some indicators (i.e., FPDS, TBED) are perfectly co-linear with the set of industry and state fixed effects. Hence, in subsequent models, we directly assess these indicators with alternative specifications. For all, we estimate regressions with robust standard errors.

To reiterate, our core research question focuses on an endogenous process. We cannot discern causality. Rather, the validity of this design lies with sampling and model specification. To understand the antecedent factors that predict high-technology ventures to seek government engagement, it is necessary to account for the entire “at-risk” population; this comprises young (private) firms. Moreover, to parse apart whether and how various factors motivate the firm’s choice to engage with the government, it is necessary to trace an exhaustive list of external and internal factors. The data and computational demands to meet these specifications are significant, requiring extensive and precise detail. As previously discussed, we validate our source of sampling by comparing startup activity between NETS and the repository of Secretary of State databases. Moreover, we draw from proprietary (NETS and Pitchbook), administrative (SAM, FPDS, and NCSES), and public (SBA, FDIC, CDFI, David Leip Atlas, and State Science Technology Institute) resources. And we rely on a powerful server (32-core processors and 64 threads, 384 GB memory, and 32 TB storage) to construct the sample and run the analyses. We construct several measures in binary functional form for several reasons: (i) to allow for greater ease of interpretation of the regressors (i.e., estimating differential effects); and (ii) to optimize around the computational demands for a logistics model of this size and scale. Nevertheless, we examine incremental variation as well (i.e., S3, S4, and S5 Tables). All data, code, and log files are publicly available to replicate the empirical analysis (https://doi.org/10.7910/DVN/KT6136).

## Results

Table 6 reports average marginal effects from the logistic regressions (Eq. 1). We report the results for internal and external factors, respectively in Columns 1 and 2, and the full specification in Column 3. We find evidence that both internal and external factors motivate firms to express formal interest in establishing a relationship with the government, though to varying degrees. The internal factors account for greater explanatory value than external factors; this is supported both by the size of the average marginal effects and differences in the adjusted r-squared values reported in Column 1 compared to Column 2.

**Table 6:** Primary logit regression.

	(1)	(2)	(3)
URM owned (Minority or Woman)	0.0364*** (0.0008)		0.0360*** (0.0008)
Woman owned	0.0039*** (0.0006)		0.0040*** (0.0006)
Minority owned	0.0218*** (0.0006)		0.0217*** (0.0006)
Size (> 1 FTE)	−0.0232*** (0.0004)		−0.0231*** (0.0004)
Any Credit	0.0040*** (0.0010)		0.0040*** (0.0010)
Any Patent	0.0084*** (0.0012)		0.0084*** (0.0011)
Institutional		0.0013** (0.0006)	−0.0001 (0.0006)
Capital		0.0009 (0.0007)	0.0013** (0.0006)
Entrepreneurial		−0.0020*** (0.0005)	−0.0026*** (0.0004)
Institutional * Capital		−0.0010 (0.0010)	−0.0006 (0.0009)
Capital * Entrepreneurial		−0.0041*** (0.0010)	−0.0020** (0.0009)
Institutional * Entrepreneurial		−0.0017** (0.0008)	−0.0008 (0.0008)
Institutional * Capital * Entrepreneurial		0.0038*** (0.0014)	0.0025* (0.0013)
Political Alignment		−0.0022*** (0.0004)	−0.0004 (0.0003)
Democratic County		0.0012*** (0.0004)	0.0000 (0.0003)
PTAC		0.0029*** (0.0004)	0.0003 (0.0003)
CDFI		−0.0043*** (0.0005)	−0.0025*** (0.0005)
Observations	1,014,868	1,011,391	1,011,391
Log psueudolikelihood	−76545.695	−98002.037	−75790.446
r2_p	0.3172	0.1175	0.3175
wald	62375.48	23206.57	61718.76
State, Industry, and Year Fixed Effects	Y	Y	Y

Dependent variable: SAM entry by firm age 3. Average marginal effects of logit model reported. Detail on dependent variable and regressors reported in Table 3. Robust standard errors in parentheses. *** p < 0.01, ** p < 0.05, * p < 0.1.

Factors that increase the likelihood of firms to register in SAM include: URM-owned firms, smaller firms, those with credit, and those with patents. The coefficient for Size is negative. Larger firm size decreases the likelihood of firm-government engagement. Interpreted another way, smaller firm size increases the likelihood of firm-government engagement. As for external factors, entrepreneurial intensity reports the most prevalent negative effect, which suggests that greater entrepreneurial intensity substitutes for firm-government engagement. Though, the triple interaction accounting for all three ecosystem indicators is positive (albeit weakly significant and economically small). In the fully specified model, we report no effect for political context and a negative effect for CDFI access.

Probing the model further, we adjust the timing specification of the dependent variable by extending entry into SAM by firm age four and five. We report consistent results (S2 Table). Separately, we adjust the functional form of the regressors. First, rather than defining the ecosystem indicators based on median trends (refer to Table 3), we identify leading and lagging activity based on 25^th^ and 75^th^ percentile distributions of distance. We report the descriptive statistics in S3 Table (along with detail on the construction of the metrics) and regression results in S4 Table. We uncover interesting trends. Note, leading values (reported in Column 2 in S4 Table) define firms with closer distance to the external measures; lagging values (reported in Column 3 in S4 Table) define those with further distance. Ventures with especially close access to external resources report a complementary relationship with institutional intermediaries yet a substitutive relationship with entrepreneurial intensity. However, more limited access to capital infrastructure and entrepreneurial intensity (i.e., further access and distance) increases the likelihood for firms to engage with the government. Importantly, the diverging trends for entrepreneurial intensity reveal the same insight (i.e., offering “two sides of the same coin”). In short, entrepreneurial intensity serves as a substitute driver of firm-government engagement – when entrepreneurial intensity is accessible, ventures are less likely to seek government engagement, and when not accessible, they are more likely to do so.

Second, we estimate the primary regression with raw values of the regressors (refer to Table 5 for descriptive statistics of the raw measures). Generally, the results are consistent to main model specification, though the regression results reported in S5 Table report greater nuance for interpretation of marginal effects.

### Heterogeneity analysis

We report heterogeneity analyses in Tables 7 and 8. The former examines heterogeneity based on internal factors, while the latter accounts for external factors. In Table 7, we split the sample by firm-ownership (i.e., non-URM, URM, woman, and minority-owned; Columns 2–5) and firm size (i.e., small vs. medium; Columns 6–7) and then re-estimate the primary specification across various sub-samples. For ease of reference, we re-report the primary results in Column 1.

**Table 7:** Heterogeneity analysis (internal).

	(1)	(2)	(3)	(4)	(5)	(6)	(7)
URM owned (Minority or Woman)	0.0358*** (0.0008)					0.0346*** (0.0008)	0.0493*** (0.0045)
Woman owned	0.0040*** (0.0006)		0.0208*** (0.0046)		0.0256*** (0.0067)	0.0035*** (0.0006)	0.0102*** (0.0038)
Minority owned	0.0216*** (0.0006)		0.1802*** (0.0041)	0.1555*** (0.0036)		0.0207*** (0.0006)	0.0313*** (0.0037)
Size (> 1 FTE)	−0.0230*** (0.0004)	−0.0210*** (0.0003)	−0.0825*** (0.0037)	−0.0801*** (0.0038)	−0.0777*** (0.0071)	−0.0224*** (0.0004)	
Any Credit	0.0040*** (0.0010)	0.0051*** (0.0007)	−0.0345*** (0.0113)	−0.0124 (0.0118)	−0.0873*** (0.0209)	0.0017* (0.0010)	0.0178*** (0.0036)
Any Patent	0.0084*** (0.0011)	0.0055*** (0.0007)	0.0220 (0.0171)	0.0243 (0.0192)	0.0245 (0.0326)	0.0073*** (0.0013)	0.0124*** (0.0038)
Institutional	−0.0004 (0.0006)	−0.0003 (0.0004)	−0.0006 (0.0067)	0.0080 (0.0069)	−0.0020 (0.0132)	−0.0003 (0.0006)	−0.0024 (0.0031)
Capital	0.0011* (0.0006)	0.0009* (0.0005)	−0.0035 (0.0082)	0.0058 (0.0084)	−0.0173 (0.0166)	0.0003 (0.0007)	0.0054* (0.0031)
Entrepreneurial	−0.0027*** (0.0004)	−0.0021*** (0.0003)	−0.0110** (0.0052)	−0.0197*** (0.0055)	−0.0021 (0.0107)	−0.0028*** (0.0004)	−0.0024 (0.0027)
Institutional * Capital	−0.0005 (0.0009)	0.0001 (0.0007)	−0.0064 (0.0114)	−0.0221* (0.0118)	0.0078 (0.0224)	−0.0000 (0.0009)	−0.0045 (0.0047)
Capital * Entrepreneurial	−0.0017* (0.0009)	−0.0008 (0.0007)	−0.0159 (0.0116)	−0.0078 (0.0120)	−0.0358 (0.0232)	−0.0013 (0.0009)	−0.0043 (0.0047)
Institutional * Entrepreneurial	−0.0005 (0.0008)	−0.0006 (0.0006)	−0.0046 (0.0092)	−0.0056 (0.0097)	−0.0099 (0.0179)	−0.0006 (0.0008)	−0.0006 (0.0048)
Institutional * Capital * Entrepreneurial	0.0023* (0.0013)	0.0013 (0.0010)	0.0215 (0.0159)	0.0209 (0.0166)	0.0298 (0.0310)	0.0019 (0.0013)	0.0064 (0.0071)
Political Alignment	−0.0002 (0.0003)	−0.0003 (0.0003)	0.0046 (0.0040)	0.0070* (0.0041)	0.0109 (0.0084)	−0.0002 (0.0003)	0.0002 (0.0019)
Democratic leaning, FIPS	−0.0001 (0.0003)	−0.0003 (0.0003)	0.0001 (0.0041)	−0.0013 (0.0042)	−0.0088 (0.0083)	−0.0003 (0.0003)	0.0033* (0.0019)
PTAC	0.0001 (0.0003)	−0.0002 (0.0003)	0.0066* (0.0039)	0.0071* (0.0041)	0.0106 (0.0081)	0.0002 (0.0003)	−0.0008 (0.0019)
CDFI	−0.0023*** (0.0005)	−0.0018*** (0.0004)	−0.0127** (0.0059)	−0.0092 (0.0061)	−0.0222* (0.0128)	−0.0022*** (0.0005)	−0.0031 (0.0026)
URM Venture Intensity	0.0015*** (0.0003)	0.0014*** (0.0002)	−0.0062 (0.0042)	−0.0081* (0.0043)	−0.0023 (0.0086)	0.0017*** (0.0003)	−0.0010 (0.0017)
SAM Venture Intensity	0.0027*** (0.0004)	0.0021*** (0.0003)	0.0134** (0.0052)	0.0174*** (0.0053)	0.0078 (0.0115)	0.0025*** (0.0004)	0.0049** (0.0023)
Sample Adjustment	Primary w/ additional controls	URM = 0	URM = 1	Woman = 1	Minority = 1	Small Biz = 1	Medium Biz = 1
Observations	1,011,391	956,135	55,196	44,197	20,693	955,981	55,369
r2_p	0.3178	0.1747	0.1552	0.161	0.0512	0.3307	0.2095
State, Industry, and Year Fixed Effects	Y	Y	Y	Y	Y	Y	Y

Dependent variable: SAM entry by firm age 3. Average marginal effects of logit model reported. Details on regressors reported in Table 3. Adjustments to sample based on URM (Minority and/or Women) designation for columns 2–5. Adjustments to sample based on size of firm (Small Biz < 5 FTE; Medium Biz 6–10 FTE) reported in columns 6 and 7. Robust standard errors in parentheses. *** p < 0.01, ** p < 0.05, * p < 0.1.

**Table 8:** Heterogeneity analysis (external).

	(1)	(2)	(3)	(4)
URM owned (Minority or Woman)	0.0360*** (0.0008)	0.0354*** (0.0008)	0.0509*** (0.0012)	0.0326*** (0.0011)
Woman owned	0.0040*** (0.0006)	0.0041*** (0.0006)	0.0045*** (0.0010)	0.0030*** (0.0010)
Minority owned	0.0217*** (0.0006)	0.0220*** (0.0006)	0.0286*** (0.0009)	0.0216*** (0.0009)
Size (> 1 FTE)	−0.0231*** (0.0004)	−0.0229*** (0.0004)	−0.0333*** (0.0006)	−0.0199*** (0.0006)
Any Credit	0.0040*** (0.0010)	0.0038*** (0.0010)	0.0035** (0.0017)	0.0043*** (0.0012)
Any Patent	0.0084*** (0.0011)	0.0087*** (0.0012)	0.0107*** (0.0019)	0.0037* (0.0021)
Institutional	−0.0001 (0.0006)	0.0001 (0.0006)	−0.0008 (0.0009)	−0.0007 (0.0012)
Capital	0.0013** (0.0006)	0.0011* (0.0006)	0.0010 (0.0010)	0.0026* (0.0014)
Entrepreneurial	−0.0026*** (0.0004)	−0.0023*** (0.0004)	−0.0040*** (0.0007)	−0.0004 (0.0007)
Institutional * Capital	−0.0006 (0.0009)	−0.0004 (0.0009)	−0.0006 (0.0015)	−0.0004 (0.0021)
Capital * Entrepreneurial	−0.0020** (0.0009)	−0.0021** (0.0009)	−0.0040*** (0.0015)	−0.0030* (0.0016)
Institutional * Entrepreneurial	−0.0008 (0.0008)	−0.0011 (0.0008)	−0.0010 (0.0013)	0.0001 (0.0014)
Institutional * Capital * Entrepreneurial	0.0025* (0.0013)	0.0026* (0.0013)	0.0050** (0.0021)	0.0026 (0.0024)
Political Alignment	−0.0004 (0.0003)	−0.0003 (0.0003)	0.0000 (0.0005)	0.0011* (0.0006)
Democratic leaning, FIPS	0.0000 (0.0003)	−0.0001 (0.0003)	−0.0008 (0.0005)	
PTAC	0.0003 (0.0003)	0.0004 (0.0003)	0.0007 (0.0005)	0.0007 (0.0005)
CDFI	−0.0025*** (0.0005)	−0.0024*** (0.0005)	−0.0039*** (0.0008)	−0.0035*** (0.0007)
Sample Adjustment	Primary	TBED = 1	FPDS = 1	TRUMP = 1
Observations	1,011,391	961,732	539,437	364,931
r2_p	0.3175	0.3162	0.3102	0.3329
State, Industry, and Year Fixed Effects	Y	Y	Y	Y

Dependent variable: SAM entry by firm age 3. Average marginal effects of logit model reported. Detail on regressors reported in Table 3. Adjustments to sample based on external factors including State TBED policy (col. 2); FPDS (col. 3); and the first Trump presidential administration (col. 4). Robust standard errors in parentheses. *** p < 0.01, ** p < 0.05, * p < 0.1.

Focusing first on the features of firm-ownership, we report some divergence and similarity of trends between non-URM and the latter set. Namely, the results for *Any Credit* report diverging trends between the two sets. In line with the primary results, non-URM owned firms report a positive, albeit economically small, effect for this measure of firm growth on the likelihood of initiating firm-government engagement, whereas we report a larger negative effect for URM and minority-owned firms. The latter set of results suggests a substitutive relationship between firm capability and firm-government engagement for historically under-served firms. Contrastingly, we report a positive effect for the measure of any patenting for non-URM owned firms and fail to report an effort for the latter set. Across all groups, we observe a similar negative effect for *Size* across all sample specifications with the largest magnitude reported for URM, woman, and minority-owned firms. In short, firm resource constraints appear to motivate such engagement regardless of ownership type. Additionally, we include two indicators – *URM Venture Intensity* and *SAM Venture Intensity* – to assess the extent to which greater concentration of local URM or SAM concentration by zip code affects such engagement. Most prominently, we report consistent positive results for SAM Venture Intensity.

Turning to features of firm size (reported in Columns 6 and 7 in Table 7), we bifurcate the sample by small (FTE < 5) and medium (FTE 6–10) sized business. Generally, we report consistent results across each sample specification. Though, we report some divergence for certain external factors. For medium-sized firms, access to capital infrastructure and, separately, location in a democratic leaning county (weakly) increase firm-engagement. The substitutive effects for entrepreneurial intensity and proximity to CDFI are pronounced for small firms.

In Table 8, we exploit geographic, industrial, and temporal features. (We are unable to include these in the primary model, Eq. 1, as they are perfectly co-linear with the set of dummies.) Namely, we split the sample by those states with TBED policies (Column 2), by industries with leading investments in federal contracting (FPDS, Column 3), and by time, distinguishing firms founded during the first Trump administration (Column 4). Again, Column 1 reports the primary results for ease of reference.

The results among the set of internal factors are consistent to the primary specification. We observe some divergence across the set of external factors. For example, high-tech ventures located in states with TBED policies or those that are founded during the first Trump administration report positive results for capital infrastructure. Whereas the substitutive effects of entrepreneurial intensity are prevalent for high-tech ventures located in states with TBED policies or those that operate in industries most salient to federal contracting priorities.

And as a final extension, we assess these external factors more directly in a separate model. Rather than estimating the primary model with a stratification technique (as reported in Table 8), we remove the set of state, industry, and year fixed effects to then include the indicators of FPDS, TBED, and Trump administration as regressors (again, without removing the fixed effects, we face the issue of perfect co-linearity). S6 Table reports these results, highlighting the complementary effect of FPDS and substitutive effect of the Trump administration. Regarding the former, high-tech ventures in industries that are more salient to federal contracting initiatives are more likely to engage with the government, while ventures founded during the first Trump administration are less likely to pursue such engagement. The remaining set of results are generally consistent to the primary model.

## Discussion

This study seeks to investigate a key underlying and overlooked assumption in the literature – the firm’s strategic choice of whether or not to engage with the government. Prior scholarship most often references this assumption as they examine subsequent activity – namely, the estimation of innovative and commercial returns from various government programs [14,20–28]. We redirect attention to unpack this core prior assumption along specific observable but heretofore largely unexplored dimensions.

Generally, the results indicate that some factors complement the firm’s choice to engage with the government, while other factors counteract or substitute for such behavior. The most prominent complementary effects include: (i) firms with URM owners; (ii) small firms; and (iii) firms with greater early-stage growth potential (measured by credit and patent activity). Conversely, the substitutive effects most consistently include: (i) firms located in more intensive entrepreneurial settings; and (ii) firms located near community development financial institutions (CDFIs). Moreover, high-tech ventures operating in industries that are more salient to federal contracting initiatives are more likely to engage with the government, while ventures founded during the first Trump administration are less likely to pursue such engagement.

To place these results in context, it is important to consider the following. There is a significant gap for new firms between establishing the startup, developing the first prototype product or service, and securing the first significant revenues [119]. Often, a firm requires significant financial capital that is too risky for private investors. To fill this gap and correct private market capital inefficiencies, the government offers a variety of programs to support early-stage technology ventures. Government awards in the form of non-equity, non-dilutive R&D funds are designed to attract (crowd in) future private investment because they reduce scientific uncertainty and often serve as valuable validation for investors [120]. At the same time, government programs advance national interest missions (i.e., space, defense, public health, etc.) and seed robust national innovation ecosystems [4,51]. We report that firms facing resource constraints are more likely to seek such support as they navigate their early stages and overcome these constraints [45].

However, if we examine the results closely, the internal motivations to seek government support are not uniform across firms; this is especially apparent from the results reported in Table 7 (contrasting non-URM from URM-owned firms). From one vantage point, we report that new firms with growth aspirations – as indicated by reaching early-stage patent milestones and seeking to obtain financial credit – motivate the choice to engage with the government. Yet from another vantage point, we report that new firms owned by minoritized groups (i.e., URM-owned firms) seek such engagement. Notably, this set of firms reports contrasting trends around growth (and the results for patenting are inconclusive).

On the one hand, the former set of results suggest that young firms turn to the government to alleviate uncertainty in their R&D pursuits. Again, it is well documented that R&D demands are inherently risky and under-supported in the private sector [45,100]. In turn, our results capture a strategic choice whereby owners of young firms rely on the government in these early stages as they traverse the infamous “valleys of death” [38].

On the other hand, the results for the set of young firms owned by minoritized groups reveal a different set of motivations. A separate stream of literature highlights the detrimental role of institutional barriers placing owners from minoritized groups at a disadvantage [115,121,122]. While these owners also seek government engagement, their motivations critically differ, whereby the indicators of growth aspirations substitute for the choice to engage with the government. Importantly, firm owners across the population face different (and contrasting) constraints and motivations as they elect to engage with the government. These diverging results offer important implications for scholars, managers, and policymakers, which we explain below.

For scholars interested in examining the returns from government programs, it is essential to account for this variation in the research design. Scholars tend to rely on quasi-experimental designs to approximate causal estimates for the returns from government programs [14,20–28]. Our results offer new guidance around firm selection. Given that the motivations to seek government support are not uniform, it is critical that scholars account for this nuance and divergence as they construct plausible counterfactual samples and define appropriate model specifications.

That said, we emphasize that our analysis offers correlative, rather than causal, conclusions. Again, our research question addresses the core prior assumption of electing to engage with the government in the first place. In this study, we are focused on understanding an endogenous process. Contrastingly, the larger stream of scholarship to which our work offers contributions focuses on the subsequent impact of programmatic returns.

Usefully, our design begins with a population-level sample to assess antecedent factors that plausibly influence such firm behavior. We identify which firms choose (and do not choose) to engage with their government in the first place, and under which conditions they do so. By sampling from this population, our study offers guidance for appropriate public policy formulation and effective administrative oversight to stimulate and/or regulate market activity. Moreover, the results can inform outreach efforts to attract firms that can support governments’ policy objectives for their key stakeholders, including their constituents and citizenry.

More broadly, our research sheds light on the core question of *what is the appropriate role of the government when engaging with high-tech ventures*? If the primary policy objective is to address market failure [45], then the trends we uncover provide critical insight to guide proposed policy reforms. Based on our results, mapping antecedent factors to national technology needs and technology-readiness requirements could enable policymakers to adjust and revise government programs that create meaningful outcomes instead of legislating in the dark. Our research offers new insights and findings for evidence-based policymaking.

For firms’ managers and entrepreneurs, our research implications are two-fold. First, our findings imply that there may be a substantial untapped and largely overlooked opportunity for obtaining resources [123]. In the U.S., less than three percent of all high-tech ventures choose to engage with their government; and even a smaller share secure procurement [124]. Second, our findings further imply that given the overall small percentage of firms seeking such engagement, the competition for these resources from the government may be relatively low and reasonably obtainable. The federal government not only acts as input for technology ventures (in the form of grants, loans, etc.) but also as a customer (buyer) at scale through procurement [100]. This enables young firms to secure a sizable customer base and a steady revenue stream. At the same time, winning a government contract from the public market signals technical credibility of the firm and opens doors for potential commercial deals in the private market [6]. Understanding this system provides entrepreneurs and managers with actionable and repeatable playbooks to grow their firms and create a competitive advantage.

### Limitations

Our study has limitations that future research may address. Of note, our sample focused on U.S. technology ventures within the 2015–2017 timeframe. We intentionally limited the sample specifications (i.e., sampling based on founding year, ownership, and industry orientation) to identify the most inclusive set of technology ventures at risk of such engagement. Certainly, the rate of government engagement would increase if we placed *additional* restrictions around how we define the at-risk sample (hence decreasing the sample size for the denominator). As the first large-scale nationwide exploratory analysis of the factors that drive ventures to engage with their governments, we elected to identify the “at-risk” set in this most inclusive manner. Future work could adjust (and perhaps further restrict this specification) if needed. In a similar vein, future research could extend this work by examining activity outside the U.S. and across broader time periods to help generalize the results.

In addition, the dependent variable captures the initial strategic move of firms to engage with the government. Within the U.S. context, once a firm registers in SAM, this is only the first of innumerable options to engage further with the government. As a follow up step in the process, firms must maintain their registration to continue access to federal programs. Moreover, firms can elect to engage more concertedly with the government, via participating in various government programs (i.e., SBIR/STTR) or securing government procurement. While we offer a baseline to understand initial government engagement, more work remains to unpack whether these antecedent factors consistently motivate decisions around deeper involvement with the government.

## Supporting information

S1 Table Correlation matrix. (DOCX)

S2 Table Timing extensions. (DOCX)

S3 Table Additional descriptive statistics for ecosystem indicators. (DOCX)

S4 Table Sensitivity analysis – ecosystem indicators extensions. (DOCX)

S5 Table Raw measures. (DOCX)

S6 Table Extensions to model specification of fixed effects and regressors. (DOCX)

## References

[1] Florida PTAC moves strategic business solutions from small startup to multi-million dollar technology services company. Florida SBDC Network State Office. 2022. https://floridasbdc.org/success_stories/strategic-business-solutions-turns-small-startup-multi-million-dollar-technology-services-company/

[2] Kropp F, Zolin R. Technological entrepreneurship and small business innovation programs. In: Ghosh A. Impact of government policies on marketing strategies. Hyderabad, India: ICFAI University Press. 2008. 10–34.

[3] Cohen L. When can government subsidize research joint ventures? Politics, economics, and limits to technology policy. The American Economic Review. 1994;84(2):159–63.

[4] Hemmatian I, Ponzio TA, Joshi AM. Exploring the role of R&D collaborations and non-patent IP policies in government technology transfer performance: Evidence from U.S. federal agencies (1999-2016). PLoS One. 2022;17(5):e0268828. doi: 10.1371/journal.pone.0268828 35609062 PMC9128958

[5] Lanahan L, Armanios D. Does more certification always benefit a venture?. Organization Science. 2018;29(5):931–47.

[6] Lanahan L, Armanios DE, Joshi AM. Inappropriateness penalty, desirability premium: what do more certifications actually signal?. Organization Science. 2022;33(2):854–71.

[7] Lerner J. The government as venture capitalist: the long-run impact of the SBIR program. J Private Equity. 2000;3(2):55–78.

[8] Mara A. Maximizing the returns of government venture capital programs. Center for Technology and National Security Policy, National Defense University. 2011.

[9] Chang CKN, Shipp SS, Wang AJ. The Advanced Technology Program: A public-private partnership for early stage technology development. Venture Capital. 2002;4(4):363–70. doi: 10.1080/1369106022000028262

[10] Arrow K. Economic welfare and the allocation of resources for invention. The rate and direction of inventive activity: economic and social factors. Princeton (NJ): Princeton University Press. 1962. 609–26.

[11] Branscomb L, Auerswald PE. Between invention and innovation an analysis of funding for early-stage technology development. 2002. https://nist.gov

[12] Bush V. Science--the endless frontier: a report to the president on a program for postwar scientific research. Alexandria (VA): National Science Foundation. 1990.

[13] Hemmatian I, Joshi AM, Inouye TM, Robinson JA. Exploring the Effects of Discretion, Discrimination, and Oversight on the Inclusiveness of Small Business Contracting. Emerald Publishing Limited. 2021.

[14] de Rassenfosse G, Jaffe A, Raiteri E. The procurement of innovation by the U.S. government. PLoS One. 2019;14(8):e0218927. doi: 10.1371/journal.pone.0218927 31404070 PMC6690509

[15] American Association for the Advancement of Science. Historical trends in federal R&D. 2023. https://www.aaas.org/programs/r-d-budget-and-policy/historical-trends-federal-rd

[16] Gans JS, Stern S. The product market and the market for “ideas”: commercialization strategies for technology entrepreneurs. Research Policy. 2003;32(2):333–50. doi: 10.1016/s0048-7333(02)00103-8

[17] Hall BH. The financing of innovation. In: Shane S. The handbook of technology and innovation management. Hoboken (NJ): Wiley-Blackwell. 2005. 409–30.

[18] Pettigrew RI, Cooke JP. At the nexus of science, engineering, and medicine: Pasteur’s quadrant reconsidered. PNAS Nexus. 2022;1(3):pgac092. doi: 10.1093/pnasnexus/pgac092 35899068 PMC9308560

[19] Stokes DE. Pasteur’s quadrant: Basic science and technological innovation. Washington (DC): Brookings Institution Press. 2011.

[20] Rathje JM, Katila R. Enabling Technologies and the Role of Private Firms: A Machine Learning Matching Analysis. Strategy Science. 2021;6(1):5–21. doi: 10.1287/stsc.2020.0112

[21] Czarnitzki D, Hünermund P, Moshgbar N. Public procurement of innovation: evidence from a German legislative reform. International Journal of Industrial Organization. 2020;71:102620.

[22] Belenzon S, Cioaca LC. Guaranteed markets and corporate scientific research. Cambridge (MA): National Bureau of Economic Research. 2021.

[23] Bruce JR, de Figueiredo JM, Silverman BS. Public contracting for private innovation: Government capabilities, decision rights, and performance outcomes. Strategic Management Journal. 2019;40(4):533–55.

[24] Myers KR, Lanahan L. Estimating Spillovers from Publicly Funded R&D: Evidence from the US Department of Energy. American Economic Review. 2022;112(7):2393–423. doi: 10.1257/aer.20210678

[25] Cohen L, Malloy CJ. Mini west Virginias: corporations as government dependents. In: https://ssrn.com/abstract=27588352016

[26] Goldfarb B. The effect of government contracting on academic research: Does the source of funding affect scientific output?. Research Policy. 2008;37(1):41–58. doi: 10.1016/j.respol.2007.07.011

[27] Josephson BW, Lee JY, Mariadoss BJ, Johnson JL. Uncle Sam rising: performance implications of business-to-government relationships. Journal of Marketing. 2019;83(1):51–72.

[28] Karlson N, Sandström C, Wennberg K. Bureaucrats or markets in innovation policy?–a critique of the entrepreneurial state. The Review of Austrian Economics. 2021;34:81–95.

[29] Abdurakhmonov M, Ridge JW, Hill AD. Unpacking firm external dependence: How government contract dependence affects firm investments and market performance. Academy of Management Journal. 2021;64(1):327–50.

[30] Howell ST. Financing innovation: Evidence from R&D grants. American Economic Review. 2017;107(4):1136–64.

[31] Azoulay P, Li D, Zivin JSG, Sampat BN. Public R&D Investments and Private-sector Patenting: Evidence from NIH Funding Rules. Rev Econ Stud. 2019;86(1):117–52. doi: 10.1093/restud/rdy034 31662587 PMC6818650

[32] Stinchcombe AL. Social structure and organizations. In: Baum JAC, Dobbin F. Economics meets sociology in strategic management. Leeds, United Kingdom: Emerald Group Publishing Limited. 2000. 229–59.

[33] Bruderl J, Schussler R. Organizational mortality: The liabilities of newness and adolescence. Administrative science quarterly. 1990;35(3):530–47.

[34] Gimenez-Fernandez EM, Sandulli FD, Bogers M. Unpacking liabilities of newness and smallness in innovative start-ups: Investigating the differences in innovation performance between new and older small firms. Research Policy. 2020;49(10):104049. doi: 10.1016/j.respol.2020.104049

[35] Schoppe LA, Chylla RW. Collaborating with universities and government labs. Research-Technology Management. 2016;59(1):67–71.

[36] Bartz W, Winkler A. Flexible or fragile? The growth performance of small and young businesses during the global financial crisis—Evidence from Germany. Journal of Business Venturing. 2016;31(2):196–215.

[37] Khaire M. Young and no money? Never mind: The material impact of social resources on new venture growth. Organization Science. 2010;21(1):168–85.

[38] Auerswald PE, Branscomb LM. Valleys of death and Darwinian seas: Financing the invention to innovation transition in the United States. The Journal of Technology Transfer. 2003;28(3–4):227–39.

[39] David PA, Hall BH, Toole AA. Is public R&D a complement or substitute for private R&D? A review of the econometric evidence. Research policy. 2000;29(4–5):497–529.

[40] Lanahan L, Graddy-Reed A, Feldman MP. The Domino Effects of Federal Research Funding. PLoS One. 2016;11(6):e0157325. doi: 10.1371/journal.pone.0157325 27327509 PMC4915724

[41] Feldman M, Fleming L, Heaton S, Desai S, Teece D. Uncommon methods and metrics for local entrepreneurial ecosystems. Research Policy. 2022;51(9):104583.

[42] Yuan X, Hao H, Guan C, Pentland A. Which factors affect the performance of technology business incubators in China? An entrepreneurial ecosystem perspective. PLoS One. 2022;17(1):e0261922. doi: 10.1371/journal.pone.0261922 35015766 PMC8752008

[43] Audretsch DB, Belitski M, Cherkas N. Entrepreneurial ecosystems in cities: The role of institutions. PLoS One. 2021;16(3):e0247609. doi: 10.1371/journal.pone.0247609 33684163 PMC7939368

[44] Joshi AM, Inouye TM, Robinson JA. How does agency workforce diversity influence federal R&D funding of minority and women technology entrepreneurs? An analysis of the SBIR and STTR programs, 2001–2011. Small Business Economics. 2018;50(3):499–519.

[45] Innovation: market failures and public policies. Handbook of Industrial Organization. Elsevier. 2021. 281–388. doi: 10.1016/bs.hesind.2021.11.013

[46] Grigoriou K, Rothaermel FT. Organizing for knowledge generation: internal knowledge networks and the contingent effect of external knowledge sourcing. Strategic Management Journal. 2016;38(2):395–414. doi: 10.1002/smj.2489

[47] Pearce JA. The relationship of internal versus external orientations to financial measures of strategic performance. Strategic Management Journal. 1983;4(4):297–306.

[48] Lee C, Lee K, Pennings JM. Internal capabilities, external networks, and performance: a study on technology‐based ventures. Strategic Management Journal. 2001;22(6–7):615–40.

[49] Stam E. Entrepreneurial ecosystems and regional policy: a sympathetic critique. European Planning Studies. 2015;23(9):1759–69.

[50] Stam E, van de Ven A. Entrepreneurial ecosystem elements. Small Bus Econ. 2019;56(2):809–32. doi: 10.1007/s11187-019-00270-6

[51] Johnson E, Hemmatian I, Lanahan L, Joshi AM. A Framework and Databases for Measuring Entrepreneurial Ecosystems. Research Policy. 2022;51(2):104398. doi: 10.1016/j.respol.2021.104398

[52] Spigel B, Harrison R. Toward a process theory of entrepreneurial ecosystems. Strategic Entrepreneurship. 2018;12(1):151–68. doi: 10.1002/sej.1268

[53] Armanios DE, Lanahan L, Yu D. Varieties of Local Government Experimentation: U.S. State-Led Technology-Based Economic Development Policies, 2000–2015. AMD. 2020;6(2):266–99. doi: 10.5465/amd.2018.0014

[54] Audretsch DB, Cunningham JA, Kuratko DF, Lehmann EE, Menter M. Entrepreneurial ecosystems: economic, technological, and societal impacts. J Technol Transf. 2019;44(2):313–25. doi: 10.1007/s10961-018-9690-4 30956392 PMC6422980

[55] Armanios DE, Eesley CE, Li J, Eisenhardt KM. How entrepreneurs leverage institutional intermediaries in emerging economies to acquire public resources. Strategic Management Journal. 2016;38(7):1373–90. doi: 10.1002/smj.2575

[56] Ierapetritis DG. Discussing the Role of Universities in Fostering Regional Entrepreneurial Ecosystems. Economies. 2019;7(4):119. doi: 10.3390/economies7040119

[57] Miller DJ, Acs ZJ. The campus as entrepreneurial ecosystem: the University of Chicago. Small Business Economics. 2017;49:75–95.

[58] Cao Z, Shi X. A systematic literature review of entrepreneurial ecosystems in advanced and emerging economies. Small Business Economics. 2021;57(1):75–110.

[59] Cohen WM, Nelson RR, Walsh JP. Links and Impacts: The Influence of Public Research on Industrial R&D. Management Science. 2002;48(1):1–23. doi: 10.1287/mnsc.48.1.1.14273

[60] Cohen S. What do accelerators do? Insights from incubators and angels. Innovations: Technology, Governance, Globalization. 2013;8(3):19–25.

[61] Tartari V, Stern S. The role of universities in local entrepreneurial ecosystems. In: Frederiksberg, Denmark, 2018.

[62] Tartari V, Stern S. More than an ivory tower: The impact of research institutions on the quantity and quality of entrepreneurship. Cambridge (MA): National Bureau of Economic Research. 2021.

[63] Bedő Z, Erdős K, Pittaway L. University-centred entrepreneurial ecosystems in resource-constrained contexts. Journal of Small Business and Enterprise Development. 2020;27(7):1149–66.

[64] Shi X, Shi Y. Unpacking the process of resource allocation within an entrepreneurial ecosystem. Research Policy. 2022;51(9):104378. doi: 10.1016/j.respol.2021.104378

[65] Bonini S, Capizzi V. The role of venture capital in the emerging entrepreneurial finance ecosystem: future threats and opportunities. Venture Capital. 2019;21(2–3):137–75.

[66] Robb AM, Robinson DT. The Capital Structure Decisions of New Firms. Rev Financ Stud. 2012;27(1):153–79. doi: 10.1093/rfs/hhs072

[67] Puri M, Zarutskie R. On the life cycle dynamics of venture‐capital‐and non‐venture‐capital‐financed firms. The Journal of Finance. 2012;67(6):2247–93.

[68] Acs ZJ, Estrin S, Mickiewicz T, Szerb L. Entrepreneurship, institutional economics, and economic growth: an ecosystem perspective. Small Bus Econ. 2018;51(2):501–14. doi: 10.1007/s11187-018-0013-9

[69] Cavallo A, Ghezzi A, Balocco R. Entrepreneurial ecosystem research: present debates and future directions. Int Entrep Manag J. 2018;15(4):1291–321. doi: 10.1007/s11365-018-0526-3

[70] Clayton P, Feldman M, Montmartin B. Funding emerging ecosystems. 2019.

[71] Samila S, Sorenson O. Venture capital as a catalyst to commercialization. Research Policy. 2010;39(10):1348–60.

[72] Alcácer J, Chung W. Location Strategies and Knowledge Spillovers. Management Science. 2007;53(5):760–76. doi: 10.1287/mnsc.1060.0637

[73] Xu Y, Wang Z-C. Can the establishment of an innovative city improve the level of technological entrepreneurship?. PLoS One. 2023;18(10):e0289806. doi: 10.1371/journal.pone.0289806 37816043 PMC10564169

[74] Szerb L, Lafuente E, Horváth K, Páger B. The relevance of quantity and quality entrepreneurship for regional performance: The moderating role of the entrepreneurial ecosystem. Regional Studies. 2019;53(9):1308–20.

[75] Xie Z, Wang X, Xie L, Duan K. Entrepreneurial ecosystem and the quality and quantity of regional entrepreneurship: A configurational approach. Journal of Business Research. 2021;128:499–509.

[76] Audretsch DB, Belitski M. Towards an entrepreneurial ecosystem typology for regional economic development: The role of creative class and entrepreneurship. Regional Studies. 2021;55(4):735–56.

[77] Leendertse J, Schrijvers M, Stam E. Measure twice, cut once: Entrepreneurial ecosystem metrics. Research Policy. 2022;51(9):104336.

[78] Audretsch DB, Belitski M. Entrepreneurial ecosystems in cities: establishing the framework conditions. The Journal of Technology Transfer. 2017;42:1030–51.

[79] Baldwin CY, Bogers MLAM, Kapoor R, West J. Focusing the ecosystem lens on innovation studies. Research Policy. 2024;53(3):104949. doi: 10.1016/j.respol.2023.104949

[80] Kromidha E, Altinay L, Arici HE. The influence of politics on the governance of an entrepreneurial ecosystem in a developing country: a generative institutional discourse approach. Entrepreneurship & Regional Development. 2024;36(9–10):1257–74. doi: 10.1080/08985626.2024.2327050

[81] Sohns F, Wójcik D. The impact of Brexit on London’s entrepreneurial ecosystem: The case of the FinTech industry. Environment and Planning A: Economy and Space. 2020;52(8):1539–59.

[82] Lanahan L, Feldman MP. Multilevel innovation policy mix: A closer look at state policies that augment the federal SBIR program. Res Policy. 2015;44(7):1387–402.

[83] Colombo MG, Dagnino GB, Lehmann EE, Salmador M. The governance of entrepreneurial ecosystems. Small Business Economics. 2019;52:419–28.

[84] Wurth B, Stam E, Spigel B. Toward an entrepreneurial ecosystem research program. Entrepreneurship Theory and Practice. 2022;46(3):729–78.

[85] Department of Defense. DOD releases report on defense spending by state in fiscal year 2019. 2021. https://www.defense.gov/News/Releases/Release/Article/2470586/dod-releases-report-on-defense-spending-by-state-in-fiscal-year-2019/

[86] Mathias BD, Williams DW, Smith AR. Entrepreneurial inception: The role of imprinting in entrepreneurial action. Journal of Business Venturing. 2015;30(1):11–28.

[87] Tzabbar D, Margolis J. Beyond the startup stage: The founding team’s human capital, new venture’s stage of life, founder–CEO duality, and breakthrough innovation. Organization Science. 2017;28(5):781–964.

[88] Henrekson M, Johansson D. Gazelles as job creators: a survey and interpretation of the evidence. Small Business Economics. 2010;35:227–44.

[89] Stam E, Wennberg K. The roles of R&D in new firm growth. Small Bus Econ. 2009;33(1):77–89. doi: 10.1007/s11187-009-9183-9

[90] Gruber M, MacMillan IC, Thompson JD. Escaping the Prior Knowledge Corridor: What Shapes the Number and Variety of Market Opportunities Identified Before Market Entry of Technology Start-ups?. Organization Science. 2013;24(1):280–300. doi: 10.1287/orsc.1110.0721

[91] Theodos B, McManus S, Rajninger T. Removing barriers to participation in local and state government procurement and contracting for entrepreneurs of color. 2024. www.urban.org

[92] Tian H, Akhtar S, Qureshi NA, Iqbal S. Predictors of entrepreneurial intentions: The role of prior business experience, opportunity recognition, and entrepreneurial education. Front Psychol. 2022;13:882159. doi: 10.3389/fpsyg.2022.882159 36405165 PMC9670128

[93] Brown R, Mason C. Looking inside the spiky bits: a critical review and conceptualisation of entrepreneurial ecosystems. Small Bus Econ. 2017;49(1):11–30. doi: 10.1007/s11187-017-9865-7

[94] Pfeffer J, Salancik G. External control of organizations—resource dependence perspective. Routledge. 2015.

[95] García-Quevedo J, Pellegrino G, Vivarelli M. R&D drivers and age: are young firms different?. Research Policy. 2014;43(9):1544–56.

[96] Hillman AJ, Withers MC, Collins BJ. Resource Dependence Theory: A Review. Journal of Management. 2009;35(6):1404–27. doi: 10.1177/0149206309343469

[97] Shepherd DA, Wiklund J. Entrepreneurial small businesses: a resource-based perspective. Cheltenham, United Kingdom: Edward Elgar Publishing. 2005.

[98] Voss U, Brettel M. The Effectiveness of Management Control in Small Firms: Perspectives from Resource Dependence Theory. Journal of Small Business Management. 2014;52(3):569–87. doi: 10.1111/jsbm.12050

[99] Drees JM, Heugens PPMAR. Synthesizing and Extending Resource Dependence Theory. Journal of Management. 2013;39(6):1666–98. doi: 10.1177/0149206312471391

[100] Myers KR, Lanahan L, Johnson EE. Small Business Innovation Applied to National Needs. National Bureau of Economic Research. 2023.

[101] Echeverri-Carroll EL, Feldman MP. Chasing entrepreneurial firms. Industry and Innovation. 2019;26(5):479–507. doi: 10.1080/13662716.2018.1475220

[102] Lanahan L, Joshi AM, Johnson E. Do public R&D subsidies produce jobs? Evidence from the SBIR/STTR program. Research Policy. 2021;50(7):104286. doi: 10.1016/j.respol.2021.104286

[103] Neumark D, Wall B, Zhang J. Do Small Businesses Create More Jobs? New Evidence for the United States from the National Establishment Time Series. Review of Economics and Statistics. 2011;93(1):16–29. doi: 10.1162/rest_a_00060

[104] Roberts B, Wolf M. High-tech industries: an analysis of employment, wages, and output. Bureau of Labor Statistics. 2018. https://www.bls.gov/opub/btn/volume-7/high-tech-industries-an-analysis-of-employment-wages-and-output.htm

[105] Brown R, Rocha A. Entrepreneurial uncertainty during the Covid-19 crisis: Mapping the temporal dynamics of entrepreneurial finance. Journal of Business Venturing Insights. 2020;14:e00174.

[106] Halchin LE. Overview of the federal procurement process and resources. Washington, DC: Congressional Research Service, Library of Congress. 2006.

[107] Hmaddi O, Lanahan L, Murray A. Tracing entrepreneurial spillovers: Evidence from the U.S. State Small Business Credit initiative and Kickstarter. Research Policy. 2025;54(4):105197. doi: 10.1016/j.respol.2025.105197

[108] Alcácer J, Chung W. Location strategies for agglomeration economies. Strat Mgmt J. 2014;35(12):1749–61. doi: 10.1002/smj.2186

[109] Delgado M, Porter ME, Stern S. Clusters, convergence, and economic performance. Research Policy. 2014;43(10):1785–99.

[110] Hidalgo CA, Balland PA, Boschma R, Delgado M, Feldman M, Frenken K. The principle of relatedness. In: Unifying Themes in Complex Systems IX: Proceedings of the Ninth International Conference on Complex Systems, 2018.

[111] Diodato D, Neffke F, O’Clery N. Why do industries coagglomerate? How Marshallian externalities differ by industry and have evolved over time. Journal of Urban Economics. 2018;106:1–26.

[112] Ellison G, Glaeser EL. Geographic Concentration in U.S. Manufacturing Industries: A Dartboard Approach. Journal of Political Economy. 1997;105(5):889–927. doi: 10.1086/262098

[113] The Carnegie Classification of Institutions of Higher Education: American Council on Education. 2025. https://carnegieclassifications.acenet.edu

[114] Autor D, Dorn D, Hanson G, Majlesi K. Importing political polarization? The electoral consequences of rising trade exposure. American Economic Review. 2020;110(10):3139–83.

[115] Johnson EE, Lanahan L, Joshi AM, Hemmatian I. The Role of Community Development Financial Institutions in Supporting Inclusive Economic Development. Economic Development Quarterly. 2025;39(3):196–210. doi: 10.1177/08912424251323208

[116] Coad A, Segarra A, Teruel M. Innovation and firm growth: does firm age play a role?. Research policy. 2016;45(2):387–400.

[117] Haltiwanger J, Jarmin RS, Miranda J. Who creates jobs? Small versus large versus young. Review of Economics and Statistics. 2013;95(2):347–61.

[118] Wooldridge JM. Introductory econometrics: A modern approach. 6th ed. Boston (MA): Cengage learning. 2016.

[119] Bennett VM, Chatterji AK. The entrepreneurial process: Evidence from a nationally representative survey. Strategic Management Journal. 2023;44(1):86–116.

[120] He S, Liu J, Ying Q. Externalities of government-oriented support for innovation: evidence from the national innovative city pilot policy in China. Economic Modelling. 2023;128:106503.

[121] Bates T, Robb A. Greater access to capital is needed to unleash the local economic development potential of minority-owned businesses. Economic Development Quarterly. 2013;27(3):250–9.

[122] Fairlie R, Robb A, Robinson DT. Black and white: access to capital among minority-owned start-ups. Management Science. 2022;68(4):2377–400.

[123] Angell M. Fewer small businesses are lining up for federal contracts. Those that did booked a record $154 billion in 2021. Inc.com. 2022. https://www.inc.com/melissa-angell/the-sba-is-rolling-out-shiny-new-credit-lines-of-up-to-5-million-also-hiking-fines.html

[124] Lanahan L, Hemmatian I, Johnson E, Joshi AM. The Impact of COVID-19 on the Landscape of Young Firms with a Public Sector Orientation. Academy of Management: Briarcliff Manor, NY. 2022.

