# Supplementary material for: Drivers of firm-government engagement for technology ventures
Source: PLoS One. 2025 Oct 10;20(10):e0333710. doi: 10.1371/journal.pone.0333710 (PMC12513645; doi:10.1371/journal.pone.0333710)
Supplement: S1 Table — (DOCX) [file pone.0333710.s001.docx]

**S1 Table. Correlation Matrix**

| Panel A: Primary | | | 1 | | 2 | | 3 | | 4 | | 5 | | 6 | | 7 | | 8 | | 9 | | 10 | | 11 | | 12 | | 13 | | 14 | | 15 | | 16 | | 17 | | 18 | |  |
| --- | --- | --- | --- | --- | --- | --- | --- | --- | --- | --- | --- | --- | --- | --- | --- | --- | --- | --- | --- | --- | --- | --- | --- | --- | --- | --- | --- | --- | --- | --- | --- | --- | --- | --- | --- | --- | --- | --- | --- |
| 1 | Sam Entry | | 1.00 | |  | |  | |  | |  | |  | |  | |  | |  | |  | |  | |  | |  | |  | |  | |  | |  | |  | |  |
| 2 | URM | | 0.33 | | 1.00 | |  | |  | |  | |  | |  | |  | |  | |  | |  | |  | |  | |  | |  | |  | |  | |  | |  |
| 3 | Woman | | 0.24 | | 0.89 | | 1.00 | |  | |  | |  | |  | |  | |  | |  | |  | |  | |  | |  | |  | |  | |  | |  | |  |
| 4 | Minority | | 0.36 | | 0.60 | | 0.30 | | 1.00 | |  | |  | |  | |  | |  | |  | |  | |  | |  | |  | |  | |  | |  | |  | |  |
| 5 | Size | | -0.09 | | -0.09 | | -0.08 | | -0.06 | | 1.00 | |  | |  | |  | |  | |  | |  | |  | |  | |  | |  | |  | |  | |  | |  |
| 6 | Any Credit | | 0.01 | | 0.02 | | 0.01 | | 0.01 | | 0.02 | | 1.00 | |  | |  | |  | |  | |  | |  | |  | |  | |  | |  | |  | |  | |  |
| 7 | Any Patent | | 0.02 | | 0.00 | | 0.00 | | 0.01 | | 0.02 | | 0.01 | | 1.00 | |  | |  | |  | |  | |  | |  | |  | |  | |  | |  | |  | |  |
| 8 | Institutional (II) | | 0.02 | | 0.02 | | 0.01 | | 0.03 | | 0.05 | | 0.00 | | 0.01 | | 1.00 | |  | |  | |  | |  | |  | |  | |  | |  | |  | |  | |  |
| 9 | Capital (CI) | | 0.00 | | 0.00 | | 0.00 | | 0.01 | | 0.05 | | 0.00 | | 0.02 | | 0.39 | | 1.00 | |  | |  | |  | |  | |  | |  | |  | |  | |  | |  |
| 10 | Ent. (EI) | | -0.01 | | -0.01 | | -0.01 | | 0.01 | | 0.02 | | 0.00 | | -0.01 | | 0.09 | | 0.11 | | 1.00 | |  | |  | |  | |  | |  | |  | |  | |  | |  |
| 11 | II * CI | | 0.01 | | 0.01 | | 0.01 | | 0.02 | | 0.04 | | 0.00 | | 0.01 | | 0.68 | | 0.71 | | 0.07 | | 1.00 | |  | |  | |  | |  | |  | |  | |  | |  |
| 12 | CI * EI | | -0.01 | | -0.01 | | -0.01 | | 0.00 | | 0.04 | | 0.00 | | 0.00 | | 0.26 | | 0.68 | | 0.47 | | 0.48 | | 1.00 | |  | |  | |  | |  | |  | |  | |  |
| 13 | II * EI | | 0.01 | | 0.01 | | 0.00 | | 0.02 | | 0.05 | | 0.00 | | 0.00 | | 0.66 | | 0.27 | | 0.47 | | 0.47 | | 0.49 | | 1.00 | |  | |  | |  | |  | |  | |  |
| 14 | II * CI * EI | | 0.00 | | 0.00 | | 0.00 | | 0.01 | | 0.04 | | 0.00 | | 0.00 | | 0.48 | | 0.50 | | 0.34 | | 0.71 | | 0.73 | | 0.72 | | 1.00 | |  | |  | |  | |  | |  |
| 15 | Pol. Align | | -0.01 | | -0.01 | | -0.01 | | -0.03 | | -0.06 | | 0.00 | | 0.00 | | -0.26 | | -0.22 | | -0.09 | | -0.20 | | -0.18 | | -0.23 | | -0.18 | | 1.00 | |  | |  | |  | |  |
| 16 | Democratic | | 0.01 | | 0.01 | | 0.00 | | 0.03 | | 0.07 | | 0.00 | | 0.01 | | 0.29 | | 0.23 | | 0.08 | | 0.23 | | 0.18 | | 0.26 | | 0.20 | | -0.45 | | 1.00 | |  | |  | |  |
| 17 | PTAC | | 0.01 | | 0.01 | | 0.00 | | 0.03 | | 0.02 | | 0.00 | | 0.00 | | 0.19 | | 0.19 | | 0.18 | | 0.15 | | 0.18 | | 0.15 | | 0.12 | | -0.17 | | 0.17 | | 1.00 | |  | |  |
| 18 | CDFI | | -0.04 | | -0.03 | | -0.02 | | -0.03 | | 0.07 | | 0.02 | | 0.01 | | 0.18 | | 0.14 | | 0.03 | | 0.15 | | 0.10 | | 0.15 | | 0.13 | | -0.13 | | 0.21 | | 0.04 | | 1.00 | |  |
| Panel B: Raw | | | | 19 | | 20 | | 21 | | 22 | | 23 | | 24 | | 25 | | 26 | | 27 | | 28 | | 29 | | 30 | | 31 | | 32 | | 33 | | 34 | | 35 | | 36 | |
| 19 | | SAM Entry | | 1.00 | |  | |  | |  | |  | |  | |  | |  | |  | |  | |  | |  | |  | |  | |  | |  | |  | |  | |
| 20 | | URM | | 0.33 | | 1.00 | |  | |  | |  | |  | |  | |  | |  | |  | |  | |  | |  | |  | |  | |  | |  | |  | |
| 21 | | Woman | | 0.24 | | 0.89 | | 1.00 | |  | |  | |  | |  | |  | |  | |  | |  | |  | |  | |  | |  | |  | |  | |  | |
| 22 | | Minority | | 0.36 | | 0.60 | | 0.30 | | 1.00 | |  | |  | |  | |  | |  | |  | |  | |  | |  | |  | |  | |  | |  | |  | |
| 23 | | Size | | -0.09 | | -0.09 | | -0.08 | | -0.06 | | 1.00 | |  | |  | |  | |  | |  | |  | |  | |  | |  | |  | |  | |  | |  | |
| 24 | | Any Credit | | 0.01 | | 0.02 | | 0.01 | | 0.01 | | 0.02 | | 1.00 | |  | |  | |  | |  | |  | |  | |  | |  | |  | |  | |  | |  | |
| 25 | | Any Patent | | 0.02 | | 0.00 | | 0.00 | | 0.01 | | 0.02 | | 0.01 | | 1.00 | |  | |  | |  | |  | |  | |  | |  | |  | |  | |  | |  | |
| 26 | | Uni dist. | | -0.02 | | -0.02 | | -0.01 | | -0.03 | | -0.08 | | -0.01 | | -0.01 | | 1.00 | |  | |  | |  | |  | |  | |  | |  | |  | |  | |  | |
| 27 | | PTAC dist. | | -0.01 | | -0.01 | | 0.00 | | -0.03 | | -0.02 | | 0.00 | | 0.00 | | 0.19 | | 1.00 | |  | |  | |  | |  | |  | |  | |  | |  | |  | |
| 28 | | Accl. dist. | | -0.01 | | -0.01 | | -0.01 | | -0.02 | | -0.03 | | 0.00 | | -0.01 | | 0.26 | | 0.35 | | 1.00 | |  | |  | |  | |  | |  | |  | |  | |  | |
| 29 | | FDIC dist. | | 0.01 | | 0.00 | | 0.01 | | -0.01 | | -0.05 | | 0.00 | | -0.01 | | 0.18 | | 0.19 | | 0.24 | | 1.00 | |  | |  | |  | |  | |  | |  | |  | |
| 30 | | CDFI dist. | | 0.04 | | 0.03 | | 0.02 | | 0.03 | | -0.06 | | -0.02 | | -0.01 | | 0.09 | | 0.06 | | 0.15 | | 0.09 | | 1.00 | |  | |  | |  | |  | |  | |  | |
| 31 | | Angle dist. | | -0.01 | | -0.01 | | 0.00 | | -0.02 | | -0.03 | | 0.00 | | -0.01 | | 0.30 | | 0.37 | | 0.73 | | 0.28 | | 0.15 | | 1.00 | |  | |  | |  | |  | |  | |
| 32 | | VC dist. | | -0.01 | | -0.01 | | 0.00 | | -0.02 | | -0.03 | | 0.00 | | -0.01 | | 0.27 | | 0.39 | | 0.65 | | 0.29 | | 0.18 | | 0.67 | | 1.00 | |  | |  | |  | |  | |
| 33 | | HHI | | 0.00 | | 0.01 | | 0.01 | | 0.01 | | -0.03 | | 0.00 | | -0.01 | | -0.07 | | -0.10 | | -0.14 | | -0.09 | | -0.09 | | -0.14 | | -0.15 | | 1.00 | |  | |  | |  | |
| 34 | | Startup Ratio | | -0.02 | | -0.03 | | -0.03 | | -0.01 | | -0.04 | | -0.01 | | -0.01 | | 0.16 | | -0.17 | | -0.11 | | -0.07 | | 0.02 | | -0.13 | | -0.19 | | 0.07 | | 1.00 | |  | |  | |
| 35 | | Pol. Align | | -0.01 | | -0.01 | | -0.01 | | -0.03 | | -0.06 | | 0.00 | | 0.00 | | 0.17 | | 0.17 | | 0.26 | | 0.18 | | 0.12 | | 0.26 | | 0.25 | | -0.08 | | -0.01 | | 1.00 | |  | |
| 36 | | Democratic | | 0.01 | | 0.01 | | 0.00 | | 0.03 | | 0.07 | | 0.00 | | 0.01 | | -0.25 | | -0.16 | | -0.27 | | -0.19 | | -0.20 | | -0.27 | | -0.26 | | 0.09 | | -0.05 | | -0.45 | | 1.00 | |

Notes: Panel A reports correlation matrix of primary measures. We report the following abbreviations: Institutional Infrastructure (II); Capital Infrastructure (CI); and Entrepreneurial Intensity (EI). Refer to Table 3 for list of the dependent variable, internal regressors, and external regressors. Panel B reports correlation matrix of raw measures. Refer to Table 5 for additional detail on raw measures.
