# Supplementary material for: Drivers of firm-government engagement for technology ventures
Source: PLoS One. 2025 Oct 10;20(10):e0333710. doi: 10.1371/journal.pone.0333710 (PMC12513645; doi:10.1371/journal.pone.0333710)
Supplement: S2 Table — (DOCX) [file pone.0333710.s002.docx]

**S2 Table. Timing Extensions**

|  | (1) | (2) | (3) |
| --- | --- | --- | --- |
| URM owned (Minority or Woman) | 0.0360*** | 0.0449*** | 0.0518*** |
|  | (0.0008) | (0.0010) | (0.0014) |
| Woman owned | 0.0040*** | 0.0048*** | 0.0049*** |
|  | (0.0006) | (0.0008) | (0.0012) |
| Minority owned | 0.0217*** | 0.0274*** | 0.0293*** |
|  | (0.0006) | (0.0007) | (0.0010) |
| Size (> 1 FTE) | -0.0231*** | -0.0279*** | -0.0300*** |
|  | (0.0004) | (0.0005) | (0.0007) |
| Any Credit | 0.0040*** | 0.0031*** | 0.0023 |
|  | (0.0010) | (0.0012) | (0.0018) |
| Any Patent | 0.0084*** | 0.0118*** | 0.0190*** |
|  | (0.0011) | (0.0014) | (0.0018) |
| Institutional | -0.0001 | -0.0006 | -0.0004 |
|  | (0.0006) | (0.0007) | (0.0009) |
| Capital | 0.0013** | 0.0014* | 0.0017* |
|  | (0.0006) | (0.0008) | (0.0010) |
| Entrepreneurial | -0.0026*** | -0.0036*** | -0.0058*** |
|  | (0.0004) | (0.0005) | (0.0008) |
| Institutional * Capital | -0.0006 | 0.0006 | 0.0008 |
|  | (0.0009) | (0.0012) | (0.0014) |
| Capital * Entrepreneurial | -0.0020** | -0.0016 | -0.0018 |
|  | (0.0009) | (0.0011) | (0.0016) |
| Institutional * Entrepreneurial | -0.0008 | -0.0008 | 0.0007 |
|  | (0.0008) | (0.0010) | (0.0014) |
| Institutional * Capital * Entrepreneurial | 0.0025* | 0.0021 | -0.0006 |
|  | (0.0013) | (0.0016) | (0.0023) |
| Political Alignment | -0.0004 | -0.0008* | -0.0011** |
|  | (0.0003) | (0.0004) | (0.0005) |
| Democratic County | 0.0000 | -0.0002 | 0.0005 |
|  | (0.0003) | (0.0004) | (0.0006) |
| PTAC | 0.0003 | 0.0006 | 0.0003 |
|  | (0.0003) | (0.0004) | (0.0006) |
| CDFI | -0.0025*** | -0.0024*** | -0.0022*** |
|  | (0.0005) | (0.0006) | (0.0008) |
| DV: SAM entry by… | Firm Age 3 | Firm Age 4 | Firm Age 5 |
| Observations | 1,011,391 | 808,579 | 478,913 |
| r2_p | 0.3172 | 0.3218 | 0.3189 |
| State, Industry, and Year Fixed Effects | Y | Y | Y |

Notes: Adjustments to dependent variable: SAM entry by firm age 3 (Column 1), firm age 4 (Column 2), and firm age 5 (Column 3). Average marginal effects of logit model reported. Detail on regressors reported in Table 3. Robust standard errors in parentheses. *** p<0.01, ** p<0.05, * p<0.1
