# Supplementary material for: Drivers of firm-government engagement for technology ventures
Source: PLoS One. 2025 Oct 10;20(10):e0333710. doi: 10.1371/journal.pone.0333710 (PMC12513645; doi:10.1371/journal.pone.0333710)
Supplement: S3 Table — (DOCX) [file pone.0333710.s003.docx]

**S3 Table. Additional Descriptive Statistics for Ecosystem Indicators**

|  | Mean | S.D. | Control | Treat | T-stat. |  |  |
| --- | --- | --- | --- | --- | --- | --- | --- |
| *Leading Ecosystem* | | | | | | | |
| Institutional | 0.12 | 0.32 | 0.12 | 0.17 | -20.90 | *** |  |
| Capital | 0.10 | 0.30 | 0.10 | 0.11 | -3.66 | *** |  |
| Entrepreneurial | 0.06 | 0.23 | 0.06 | 0.04 | 16.68 | *** |  |
| Institutional * Capital | 0.05 | 0.22 | 0.05 | 0.07 | -10.18 | *** |  |
| Capital * Entrepreneurial | 0.01 | 0.10 | 0.01 | 0.01 | 1.96 | * |  |
| Institutional * Entrepreneurial | 0.01 | 0.09 | 0.01 | 0.01 | 6.21 | *** |  |
| Institutional * Capital * Ent. | 0.00 | 0.06 | 0.00 | 0.00 | 2.77 | *** |  |
|  |  |  |  |  |  |  |  |
| *Lagging Ecosystem* | | | | | | | |
| Institutional | 0.09 | 0.28 | 0.09 | 0.07 | 9.38 | *** |  |
| Capital | 0.10 | 0.29 | 0.10 | 0.10 | -0.81 |  |  |
| Entrepreneurial | 0.09 | 0.28 | 0.09 | 0.11 | -9.85 | *** |  |
| Institutional * Capital | 0.03 | 0.17 | 0.03 | 0.03 | 1.62 |  |  |
| Capital * Entrepreneurial | 0.02 | 0.13 | 0.02 | 0.02 | 0.17 |  |  |
| Institutional * Entrepreneurial | 0.02 | 0.14 | 0.02 | 0.02 | -3.10 | *** |  |
| Institutional * Capital * Ent. | 0.01 | 0.08 | 0.01 | 0.01 | -2.30 | ** |  |
| Observations | 1,014,868 |  | 991,265 | 23,603 |  |  |  |

Notes: **Leading Ecosystem** constructed as follows: institutional intermediary and capital infrastructure measures based on whether venture is geographically proximate to the organization (i.e., located below the 25^th^ percentile of distance); Entrepreneurial intensity measures based on leading startup ratios (i.e., above the 75^th^ percentile) and HHI indicative of greater market competition (i.e., below the 25^th^ percentile). **Lagging Ecosystem** constructed as follows: institutional intermediary and capital infrastructure measures based on whether venture is geographically distant to the organization (i.e., located above the 75^th^ percentile of distance); entrepreneurial intensity measures based on lagging startup ratios (i.e., below the 25^th^ percentile) and HHI indicative of less competition and hence greater market power and concentration (i.e., above the 75^th^ percentile). Comparison of means reports between full sample and sub-sample that register in SAM by age 3. Entrepreneurial intensity measures reported for 1,014,115 observations. *** p<0.01, ** p<0.05, * p<0.1
