# Supplementary material for: Drivers of firm-government engagement for technology ventures
Source: PLoS One. 2025 Oct 10;20(10):e0333710. doi: 10.1371/journal.pone.0333710 (PMC12513645; doi:10.1371/journal.pone.0333710)
Supplement: S4 Table — (DOCX) [file pone.0333710.s004.docx]

**S4 Table. Sensitivity Analysis – Ecosystem Indicator Extensions**

|  | (1) | (2) | (3) |
| --- | --- | --- | --- |
| Institutional | -0.0001 | 0.0015*** | -0.0009 |
|  | (0.0006) | (0.0005) | (0.0008) |
| Capital | 0.0013** | -0.0000 | 0.0014** |
|  | (0.0006) | (0.0007) | (0.0006) |
| Entrepreneurial | -0.0026*** | -0.0051*** | 0.0021*** |
|  | (0.0004) | (0.0008) | (0.0006) |
| Institutional * Capital | -0.0006 | 0.0010 | 0.0014 |
|  | (0.0009) | (0.0010) | (0.0013) |
| Capital * Entrepreneurial | -0.0020** | -0.0005 | -0.0025* |
|  | (0.0009) | (0.0029) | (0.0014) |
| Institutional * Entrepreneurial | -0.0008 | 0.0031 | -0.0022 |
|  | (0.0008) | (0.0019) | (0.0017) |
| Institutional * Capital * Entrepreneurial | 0.0025* | -0.0028 | 0.0028 |
|  | (0.0013) | (0.0041) | (0.0027) |
| Adjustments to Ecosystem Indicators and Government Training based on… | None (primary) | Leading values | Lagging values |
| Observations | 1,011,391 | 1,011,391 | 1,011,391 |
| r2_p | 0.3172 | 0.3173 | 0.3172 |
| State, Industry, and Year Fixed Effects | Y | Y | Y |

Notes: Dependent variable: SAM entry by firm age 3. Average marginal effects of logit model reported. Ecosystem indicators and government training regressors adjusted based on sample descriptive statistics. Ecosystem indicators and government training regressors for column 1 based on sample median; column 2 based on leading values; column 3 based on lagging values. Refer to notes in S2 Table for detail on ecosystem indicator regressors. For government training regressors, leading values based on proximate distance (below 25^th^ percentile of distance), and lagging values based on further distance (above 75^th^ percentile of distance. Internal and external political regressors not reported (though they are equivalent across regressions and available upon request). Robust standard errors in parentheses. *** p<0.01, ** p<0.05, * p<0.1
