# Supplementary material for: Drivers of firm-government engagement for technology ventures
Source: PLoS One. 2025 Oct 10;20(10):e0333710. doi: 10.1371/journal.pone.0333710 (PMC12513645; doi:10.1371/journal.pone.0333710)
Supplement: S5 Table — (DOCX) [file pone.0333710.s005.docx]

**S5 Table. Raw Measures**

|  | (1) | (2) | (3) |
| --- | --- | --- | --- |
| URM owned (Minority or Woman) | 0.0394*** |  | 0.0389*** |
|  | (0.0007) |  | (0.0007) |
| Woman owned | 0.0039*** |  | 0.0039*** |
|  | (0.0006) |  | (0.0006) |
| Minority owned | 0.0222*** |  | 0.0223*** |
|  | (0.0006) |  | (0.0006) |
| Employment (arcsinh) | -0.0133*** |  | -0.0132*** |
|  | (0.0004) |  | (0.0004) |
| Any Credit | 0.0047*** |  | 0.0047*** |
|  | (0.0010) |  | (0.0010) |
| Any Patent | 0.0088*** |  | 0.0086*** |
|  | (0.0012) |  | (0.0012) |
| University Distance (arcsinh) |  | -0.0007*** | 0.0002 |
|  |  | (0.0002) | (0.0002) |
| Accelerator Distance (arcsinh) |  | -0.0010*** | -0.0006*** |
|  |  | (0.0002) | (0.0002) |
| FDIC Distance (arcsinh) |  | 0.0024*** | 0.0017*** |
|  |  | (0.0002) | (0.0002) |
| Angel Distance (arcsinh) |  | 0.0006*** | 0.0002 |
|  |  | (0.0002) | (0.0002) |
| VC Distance (arcsinh) |  | -0.0008*** | -0.0011*** |
|  |  | (0.0002) | (0.0002) |
| Unconcentrated Market (HHI < 0.15) |  | 0.0014** | 0.0009 |
|  |  | (0.0006) | (0.0006) |
| Startup Ratio |  | -0.1168*** | -0.0963*** |
|  |  | (0.0078) | (0.0070) |
| Political Alignment |  | -0.0019*** | -0.0000 |
|  |  | (0.0004) | (0.0003) |
| Democratic County |  | 0.0009** | -0.0004 |
|  |  | (0.0004) | (0.0003) |
| PTAC Distance (arcsinh) |  | -0.0011*** | -0.0001 |
|  |  | (0.0002) | (0.0002) |
| CDFI Distance (arcsinh) |  | 0.0031*** | 0.0013*** |
|  |  | (0.0005) | (0.0004) |
| Observations | 1,014,868 | 1,011,391 | 1,011,391 |
| r2_p | 0.3023 | 0.1185 | 0.3035 |
| State, Industry, and Year Fixed Effects | Y | Y | Y |

Notes: Dependent variable: SAM entry by firm age 3. Average marginal effects of logit model reported. Interpret continuous measures (i.e., arcsinh) as semi-elasticity. Descriptive statistics of (raw) regressors reported in Table 5. Robust standard errors in parentheses. *** p<0.01, ** p<0.05, * p<0.1
