# Supplementary material for: Drivers of firm-government engagement for technology ventures
Source: PLoS One. 2025 Oct 10;20(10):e0333710. doi: 10.1371/journal.pone.0333710 (PMC12513645; doi:10.1371/journal.pone.0333710)
Supplement: S6 Table — (DOCX) [file pone.0333710.s006.docx]

**S6 Table.** **Extensions to Model Specification of Fixed Effects and Regressors**

|  | (1) | (2) |
| --- | --- | --- |
| URM owned (Minority or Woman) | 0.0360*** | 0.0435*** |
|  | (0.0008) | (0.0007) |
| Woman owned | 0.0040*** | 0.0009 |
|  | (0.0006) | (0.0006) |
| Minority owned | 0.0217*** | 0.0246*** |
|  | (0.0006) | (0.0005) |
| Size (> 1 FTE) | -0.0231*** | -0.0191*** |
|  | (0.0004) | (0.0003) |
| Any Credit | 0.0040*** | 0.0065*** |
|  | (0.0010) | (0.0010) |
| Any Patent | 0.0084*** | 0.0184*** |
|  | (0.0011) | (0.0012) |
| Institutional | -0.0001 | 0.0020*** |
|  | (0.0006) | (0.0006) |
| Capital | 0.0013** | 0.0011* |
|  | (0.0006) | (0.0007) |
| Entrepreneurial | -0.0026*** | -0.0039*** |
|  | (0.0004) | (0.0004) |
| Institutional * Capital | -0.0006 | 0.0005 |
|  | (0.0009) | (0.0009) |
| Capital * Entrepreneurial | -0.0020** | -0.0030*** |
|  | (0.0009) | (0.0009) |
| Institutional * Entrepreneurial | -0.0008 | 0.0026*** |
|  | (0.0008) | (0.0008) |
| Institutional * Capital * Entrepreneurial | 0.0025* | 0.0015 |
|  | (0.0013) | (0.0013) |
| Political Alignment | -0.0004 | -0.0007** |
|  | (0.0003) | (0.0003) |
| Democratic County | 0.0000 | 0.0015*** |
|  | (0.0003) | (0.0003) |
| PTAC | 0.0003 | -0.0003 |
|  | (0.0003) | (0.0003) |
| CDFI | -0.0025*** | -0.0072*** |
|  | (0.0005) | (0.0003) |
| FPDS |  | 0.0179*** |
|  |  | (0.0003) |
| State TBED Policy |  | 0.0010 |
|  |  | (0.0006) |
| Trump 1^st^ Presidential Admin. |  | -0.0013*** |
|  |  | (0.0003) |
| Observations | 1,011,391 | 1,011,395 |
| r2_p | 0.3172 | 0.2621 |
| State, Industry, and Year Fixed Effects | Y | N |

Notes: Adjustments regressors and inclusion (or not) of state, industry, and year fixed effects. Average marginal effects of logit model reported. Detail on regressors reported in Table 3. Robust standard errors in parentheses. *** p<0.01, ** p<0.05, * p<0.1
